# Comparative efficacy of non-pharmacological interventions on fatigue in people undergoing hemodialysis: a systematic review and network meta-analysis

**DOI:** 10.3389/fmed.2026.1862895

**Published:** 2026-08-19

**Authors:** Juncai Li, Zhe Meng, Jing Yang, Longjie Wei, Qirui Zhang, Yijia Lin, Bonolo William, Xiuling Zhou

**Affiliations:** 1 School of Nursing, Changchun University of Chinese Medicine, Changchun, Jilin, China; 2 The Affiliated Hospital of Changchun University of Chinese Medicine, Changchun, Jilin, China

**Keywords:** fatigue, maintenance hemodialysis, network meta-analysis, non-pharmacological interventions, quality of life, systematic review

## Abstract

**Background:**

Fatigue is a prevalent and debilitating symptom among people undergoing maintenance hemodialysis (MHD). Pharmacological options for fatigue are limited, and treatment may be complicated by altered pharmacokinetics, polypharmacy, and adverse effects. Non-pharmacological interventions have therefore received increasing attention, but their comparative efficacy remains uncertain. This study aimed to compare and rank their efficacy for fatigue relief in people undergoing MHD.

**Methods:**

PubMed, Embase, the Cochrane Library, CINAHL, and Web of Science were searched from inception to December 14, 2025. Randomized controlled trials of non-pharmacological interventions for fatigue were included. Risk of bias was assessed using the revised Cochrane risk-of-bias tool. Pairwise and frequentist random-effects network meta-analyses were performed. Treatment rankings were estimated using the surface under the cumulative ranking curve, and confidence in the evidence was evaluated using the Confidence in Network Meta-Analysis framework.

**Results:**

Thirty-nine trials involving 2,943 participants evaluated eight active interventions, sham control, and usual care. Most trials were judged to have some concerns regarding risk of bias. Compared with usual care, brief behavioral intervention (SMD = −1.73, 95% CI: −2.46 to −0.99) and mind–body therapy (SMD = −1.66, 95% CI: −2.20 to −1.12) had the largest estimated reductions in fatigue and ranked first and second, respectively. Cognitive behavioral therapy, aromatherapy, exercise, acupoint stimulation, and manual therapy were also associated with lower fatigue, whereas estimates for sham control and non-invasive brain stimulation versus usual care were not statistically significant. There was no statistical evidence of global or local inconsistency. The direction of several estimates was maintained in sensitivity analyses, although small-study effects and estimate instability remained possible; exploratory meta-regression suggested that fatigue scale type may modify treatment effects. For comparisons with usual care, confidence was moderate for brief behavioral intervention, mind–body therapy, and aromatherapy, and low or very low for the remaining interventions.

**Conclusion:**

Brief behavioral intervention and mind–body therapy appear to be the most promising options for reducing fatigue in people undergoing MHD, while aromatherapy was also supported by moderate-confidence evidence. However, these findings, particularly the treatment rankings, should be interpreted cautiously because of limited direct comparative evidence and uncertainty in the evidence base.

**Systematic review registration:**

(https://www.crd.york.ac.uk/PROSPERO/view/CRD420261355908) PROSPERO (CRD420261355908).

## 1 Introduction

Chronic kidney disease (CKD) constitutes a major global public health priority, currently affecting an estimated 850 million individuals worldwide (1, 2). For people whose CKD progresses to end-stage kidney disease (ESKD), maintenance hemodialysis (MHD) is the most commonly used kidney replacement therapy (3). Although MHD is life-sustaining, it is accompanied by a substantial symptom burden, among which fatigue is one of the most pervasive and debilitating symptoms (4). Fatigue is highly prevalent among people receiving dialysis across different countries and clinical settings, with reported prevalence estimates ranging from 60 to 97% (5). A global systematic review and meta-analysis incorporating evidence from 62 countries estimated that fatigue affected approximately 70% of people receiving dialysis (6). Focusing specifically on post-dialysis fatigue, another systematic review reported a pooled prevalence of 61.0% (7). Unlike ordinary tiredness, dialysis-related fatigue is a multidimensional syndrome encompassing physical, cognitive, and emotional exhaustion that is characteristically unrelieved by rest (8). This persistent symptom burden compromises functional capacity, treatment adherence, and health-related quality of life (5, 9), and has also been associated with cardiovascular events and mortality (10–12).

Pharmacological options for fatigue are limited, and their effects may be incomplete or inconsistent (13). In people receiving hemodialysis, long-term pharmacological management may also be complicated by altered pharmacokinetics, polypharmacy, and systemic adverse effects (14–16). Consequently, non-pharmacological interventions have received increasing attention as strategies for fatigue management (17). In recent years, a diverse range of nurse-led and multidisciplinary interventions has been investigated in renal care. These include structured physical exercise, such as intradialytic aerobic and resistance training (18), and behavioral and educational interventions, such as energy-conservation education and recreational therapy (19, 20). Intradialytic neuromuscular electrical stimulation has also been investigated as a low-effort rehabilitation strategy, with companion reports from the same trial cohort showing improvements in muscle strength, functional capacity, and postural balance (21), and in exercise capacity, neuromuscular activation, and muscle oxygenation (22), although these outcomes are distinct from patient-reported fatigue. Mind–body therapies, such as yoga, and complementary approaches, including acupoint-related therapies and aromatherapy, have also emerged as promising options for reducing fatigue (23–25). These interventions are particularly relevant to holistic nursing practice because they target potentially modifiable factors, support self-management, and may improve overall well-being (26).

Despite the growing number of randomized controlled trials (RCTs), the available evidence has largely been synthesized through pairwise meta-analyses that evaluate individual intervention categories separately (27–29). Although these reviews suggest potential benefits of specific approaches, such as exercise or acupressure (27, 29), their findings do not permit direct comparisons across the full range of available interventions. Differences in study populations, comparators, fatigue measures, and analytical methods further limit comparisons between separate reviews. In addition, conventional pairwise meta-analysis cannot estimate the relative effects of interventions that have not been directly compared within the same trials. As a result, the existing evidence does not provide a unified comparison of the relative effects of different non-pharmacological interventions, leaving uncertainty regarding which strategies may provide the greatest benefit. This limits the ability of nephrology nurses and other healthcare professionals to select individualized, evidence-informed interventions for people receiving MHD (17, 30).

Network meta-analysis (NMA) can address these limitations by integrating direct and indirect evidence and simultaneously comparing multiple interventions within a common analytical framework (31). Therefore, the present study aimed to evaluate the comparative efficacy of different non-pharmacological interventions for fatigue among people receiving MHD and to estimate their relative rankings. By providing a comprehensive synthesis of the available evidence, this study may help nephrology nurses and multidisciplinary teams select individualized fatigue-management strategies and support person-centered care.

## 2 Methods

This systematic review with NMA was conducted in accordance with the Cochrane Handbook for Systematic Reviews of Interventions (32) and reported in accordance with the PRISMA-NMA statement (33). The review was registered in PROSPERO before data extraction, risk-of-bias assessment, and data synthesis were initiated (CRD420261355908).

### 2.1 Search strategy

A systematic and comprehensive literature search of five electronic databases—PubMed, Embase, The Cochrane Library, CINAHL, and Web of Science Core Collection—was conducted from their inception to December 14, 2025. The search strategy combined controlled vocabulary (e.g., MeSH terms and CINAHL Headings) with free-text keywords and was adapted to the syntax of each database. The search strings were constructed around three primary concepts: “hemodialysis,” “fatigue,” and “non-pharmacological interventions.” To maximize the retrieval of relevant trials, the Cochrane Highly Sensitive Search Strategy for identifying randomized trials was applied where appropriate. Additionally, the reference lists of the included studies and relevant review articles were manually screened to identify any further eligible literature. Detailed search strategies for each database are provided in Supplementary Table 1.

### 2.2 Inclusion and exclusion criteria

The eligibility of studies was predetermined based on the PICOS framework.

Inclusion criteria were as follows: (1) Population: Adults aged ≥18 years receiving MHD; (2) Interventions: Trials must have evaluated at least one eligible non-pharmacological intervention classified as exercise, mind–body therapy, cognitive behavioral therapy (CBT), brief behavioral intervention, aromatherapy, manual therapy, acupoint stimulation, or non-invasive brain stimulation. The operational definitions and classifications of these interventions are detailed in Table 1; (3) Comparisons: Eligible comparators included usual care, sham control, or another active non-pharmacological intervention; (4) Outcomes: Fatigue assessed using a validated scale, such as the Fatigue Severity Scale or Piper Fatigue Scale, with sufficient quantitative data for effect-size estimation; and (5) Study design: RCTs.

**Table 1:** Operational definitions of intervention and comparator nodes.

Intervention/Comparator node	Definition
Usual care	Routine dialysis-related nursing and medical care, a waiting-list condition, or no additional active intervention specifically targeting fatigue.
Sham control	An intervention-specific control procedure designed to resemble the corresponding active intervention while omitting its hypothesized principal therapeutic component.
Exercise	Structured physical activity intended to improve physical function or fitness, including aerobic, resistance, cycling, and other intradialytic or interdialytic exercise programs.
Mind–body therapy	Interventions intended primarily to influence psychophysiological regulation, relaxation, or perceived well-being through coordinated mental and bodily processes, including yoga, progressive muscle relaxation, Benson relaxation combined with music, meditation-related practices, and Reiki (47).
CBT	Structured psychological treatment based on identifying and modifying maladaptive cognitions and behaviors through techniques such as cognitive restructuring, behavioral activation, and problem solving (77).
Brief behavioral intervention	Time-limited, comparatively low-resource behavioral or psychoeducational programs focused on fatigue self-management, coping, activity regulation, recreation, energy conservation, or sleep-related behavioral support. This node included energy-conservation education, humor therapy, recreational therapy, and sleep-hygiene education, but excluded interventions using a structured formulation-driven CBT protocol (78, 79).
Aromatherapy	Therapeutic use of essential oils through inhalation, topical application, or associated procedures, with the essential oil constituting the principal distinguishing therapeutic component (56).
Manual therapy	Interventions primarily involving manual manipulation of soft tissues or body regions, including massage and related hands-on techniques.
Acupoint stimulation	Stimulation of specified body or auricular acupoints using acupuncture, acupressure, auriculotherapy, laser acupuncture, or related methods, with acupoint stimulation constituting the principal therapeutic component.
Non-invasive brain stimulation	Non-invasive stimulation applied to the brain to modulate cortical activity; in this review, the node was represented by transcranial direct-current stimulation and excluded peripheral neuromuscular electrical stimulation.

Multicomponent interventions were classified according to their predominant therapeutic component. Study-level intervention components, final node assignments, and the rationale for each classification are provided in Supplementary Table 2.

Exclusion criteria included: (1) Conference abstracts and study protocols; (2) Studies with inaccessible full texts; and (3) Studies not published in English.

### 2.3 Literature selection and data extraction

Two independent reviewers (JL and ZM) performed study selection and data extraction. After removing duplicates, the titles and abstracts of all records were screened, followed by full-text assessment of potentially eligible articles. Any disagreements were resolved through consensus or consultation with a third reviewer (XZ).

Data were independently extracted using a standardized form, including study characteristics, participant characteristics, dialysis vintage and dialysis adequacy when reported, intervention details, and outcomes. Multicomponent interventions were independently classified by the same two reviewers according to the predominant therapeutic component; study-level components, node assignments, and rationales are provided in Supplementary Table 2, and arms assigned to the same node were combined after classification. To ensure quantitative consistency, standard errors (SEs) and 95% confidence intervals (CIs) were converted to standard deviations (SDs). Furthermore, data presented as medians with ranges or interquartile ranges were estimated as means and SDs using validated algorithms (34, 35). When a trial reported fatigue at more than one post-baseline time point, the assessment corresponding to completion of the planned intervention was selected for the primary analysis. Later post-intervention follow-up measurements were not combined with end-of-intervention outcomes.

### 2.4 Risk of bias

Two independent reviewers (JL and JY) assessed the risk of bias in the included RCTs using the revised Cochrane risk-of-bias tool (RoB 2) (36). Any discrepancies were resolved through consensus or consultation with a third reviewer (XZ). The tool assesses risk of bias across five domains: bias arising from the randomization process, bias due to deviations from intended interventions, bias due to missing outcome data, bias in measurement of the outcome, and bias in selection of the reported result. The overall risk-of-bias judgment for the fatigue outcome at the selected assessment point was classified as “low risk,” “some concerns,” or “high risk.”

### 2.5 Data synthesis and analysis

#### 2.5.1 Pairwise meta-analysis

Pairwise random-effects meta-analyses were performed using Review Manager (RevMan, version 5.4) to summarize direct evidence. Because fatigue was assessed using different scales, effects were expressed as standardized mean differences (SMDs) with 95% CIs based on post-intervention scores, with negative values favoring the intervention. Statistical heterogeneity was assessed using the *I^2^* statistic. *I^2^* values of 25, 50, and 75% were considered to indicate low, moderate, and high heterogeneity, respectively, with values greater than 50% suggesting substantial heterogeneity (37).

#### 2.5.2 Network meta-analysis

A frequentist random-effects NMA was performed using Stata software (version 17.0; StataCorp, College Station, TX, USA), with between-study heterogeneity estimated using restricted maximum likelihood. Usual care was used as the reference treatment (31). A common between-study heterogeneity variance was assumed across treatment comparisons. The transitivity assumption was evaluated by examining the clinical definitions of the nodes and the distribution of potential effect modifiers, including participant age, dialysis vintage, intervention duration, geographic region, fatigue assessment scale, and overall risk of bias, across treatment comparisons.

Network plots were generated to visualize the evidence structure, with node sizes proportional to the number of trials involving each node and edge thicknesses proportional to the number of trials contributing to each direct comparison (38). The relative ranking of interventions was summarized using the surface under the cumulative ranking curve (SUCRA) (39). SUCRA values range from 0 to 100%, with higher values indicating a greater likelihood that an intervention occupies a favorable rank. Ranking results were interpreted alongside the corresponding effect estimates, 95% CIs, network geometry, and confidence in the evidence rather than as definitive evidence of clinical superiority.

#### 2.5.3 Assessment of inconsistency

The consistency assumption was evaluated at both global and local levels. Global inconsistency was assessed using the design-by-treatment interaction model (40), while local inconsistency was examined using the node-splitting method to identify potential disagreement between direct and indirect estimates (41). A two-sided *p*-value <0.05 was considered evidence of statistically significant inconsistency. In the absence of statistically significant inconsistency, results from the consistency model were reported.

#### 2.5.4 Small-study effects, meta-regression, and sensitivity analyses

Potential small-study effects and publication bias were visually assessed using a comparison-adjusted funnel plot generated in Stata (42). Formal regression-based testing for funnel-plot asymmetry was not used because no individual direct comparison included at least 10 studies.

Exploratory univariable network meta-regression analyses were performed in separate models to investigate potential sources of heterogeneity. The examined covariates were intervention duration (≤6 weeks vs. >6 weeks), fatigue scale type, mean participant age, mean dialysis vintage, geographic region (Iran or Türkiye vs. other regions), publication year as a continuous variable, and publication period (>2021 vs. ≤2021). Publication year and publication period were examined in separate models. Models involving mean participant age and mean dialysis vintage were based on available cases, and mean participant age was centered around the network mean to facilitate interpretation.

Three sensitivity analyses were conducted by excluding (1) trials at high overall risk of bias, (2) trials with at least one analytical arm containing fewer than 20 participants, and (3) trials containing multicomponent interventions. Additional exploratory analyses examined scale distributions by network node, geographic region, and publication period and restricted the network to FSS/PFS-based trials. Because the restricted network was disconnected, the NMA was limited to its main connected component.

### 2.6 Confidence in the evidence

Confidence in the evidence for each network comparison was evaluated using the Confidence in Network Meta-Analysis (CINeMA) framework through its web application (43). CINeMA assesses six domains: within-study bias, reporting bias, indirectness, imprecision, heterogeneity, and incoherence. A prespecified equivalence range of −0.20 to 0.20 SMD units was used for judgments of imprecision, heterogeneity, and incoherence. Overall confidence was categorized as high, moderate, low, or very low.

## 3 Results

### 3.1 Study selection

**Figure 1:**
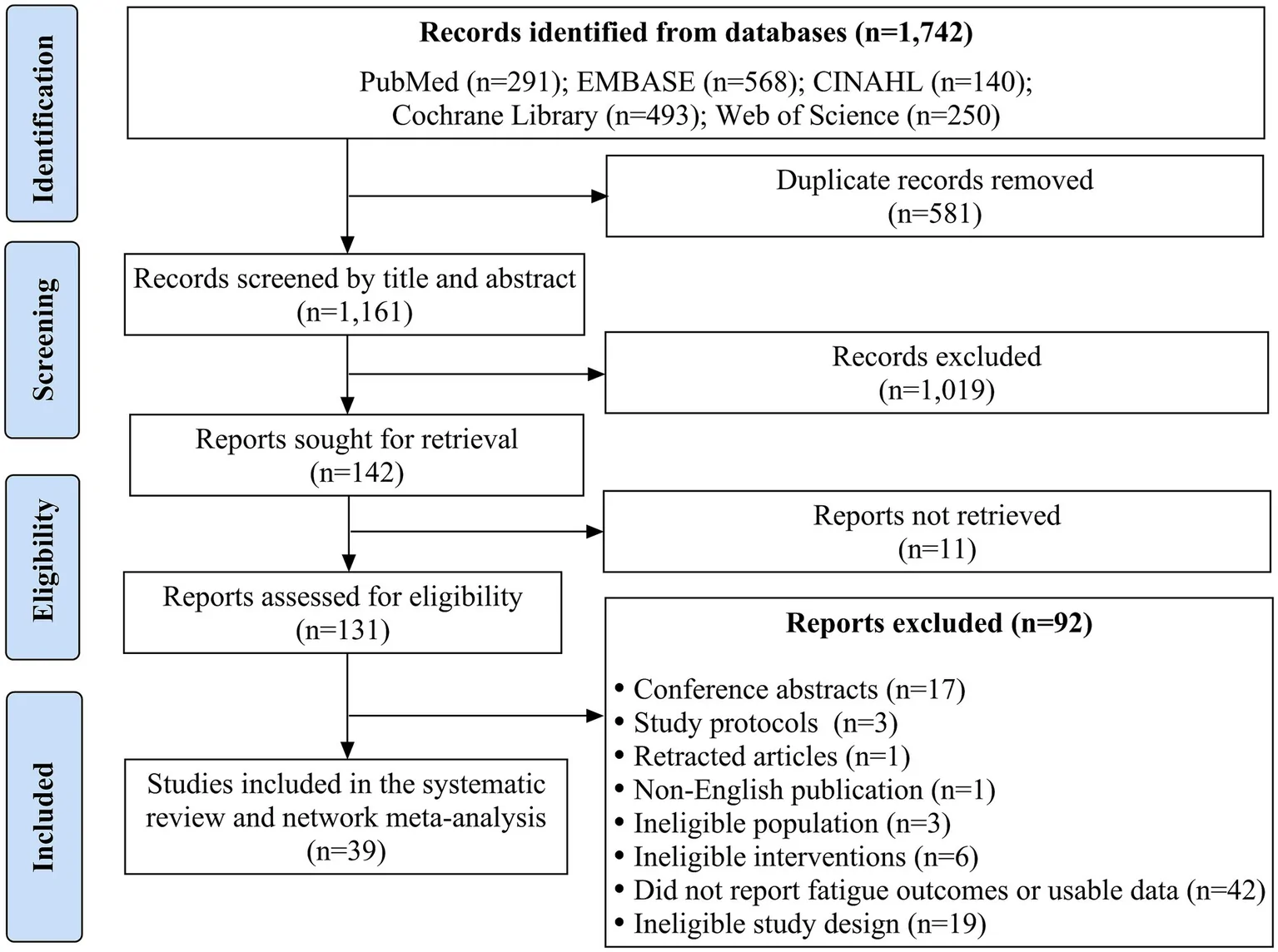
PRISMA flow diagram of study selection.

The database search identified 1,742 records. After removing 581 duplicates, 1,161 records were screened by title and abstract, of which 1,019 were excluded. Full-text reports were sought for 142 records; 11 could not be retrieved, leaving 131 reports for eligibility assessment. Ninety-two reports were excluded, and 39 RCTs were included in the systematic review and NMA. The study selection process is shown in Figure 1.

### 3.2 Characteristics of included studies

The 39 included trials, published between 2004 and 2025, comprised 2,943 participants and 81 analytical arms. After arms assigned to the same intervention node were combined, 36 trials contributed two analytical arms and three contributed three analytical arms. Mean participant age was reported in 34 trials and ranged from 39.50 to 64.90 years, while mean dialysis vintage was reported in 23 trials and ranged from 6.26 to 85.69 months. A total of 15 trials were conducted in Iran, 12 in Türkiye, 6 in China, and one each in Nepal, Brazil, Indonesia, Pakistan, the United Kingdom, and India.

Intervention duration was ≤6 weeks in 26 trials and >6 weeks in 13 trials. Fatigue was assessed using the Fatigue Severity Scale in 11 trials, the Piper Fatigue Scale in 11, the Multidimensional Fatigue Inventory in four, and other validated instruments in 13.

Seven trials required predominant-component classification (Supplementary Table 2), and scale distributions across network nodes, geographic regions, and publication periods are summarized in Supplementary Table 3.

The evidence network comprised 10 nodes. Usual care was represented in 29 trials, while the sham-control node included eight trials and 272 participants; CBT and non-invasive brain stimulation were represented by only two and one trials, respectively. Participant age and intervention duration showed broadly overlapping distributions across the more frequently studied comparisons, whereas geographic setting and fatigue-scale use were unevenly distributed and dialysis vintage was incompletely reported. Detailed study characteristics and node assignments are presented in Supplementary Table 4, and the transitivity assessment is summarized in Supplementary Table 5.

### 3.3 Risk of bias

According to the RoB 2 assessments, 2 trials were judged to have a low overall risk of bias (5.1%), 27 had some concerns (69.2%), and 10 had a high risk of bias (25.6%). Concerns most frequently arose from the randomization process and outcome measurement, particularly because fatigue was self-reported and participant blinding was generally infeasible. Detailed judgments are presented in Supplementary Figures 1, 2.

### 3.4 Pairwise meta-analysis results

Fourteen direct treatment comparisons were available. Ten comparisons included at least two trials and were pooled, whereas four were informed by a single trial. Among pooled comparisons with usual care, estimates favored sham control, exercise, mind–body therapy, brief behavioral intervention, aromatherapy, manual therapy, and acupoint stimulation, with pooled SMDs ranging from −1.92 to −0.52. Aromatherapy (SMD = −2.02, 95% CI − 3.11 to −0.94) and acupoint stimulation (SMD = −1.00, 95% CI − 1.75 to −0.25) were also associated with lower fatigue scores than sham control, whereas no clear difference was found between mind–body therapy and exercise (SMD = −0.34, 95% CI − 1.01 to 0.33). Substantial heterogeneity was observed in most pooled comparisons. Pooled pairwise estimates are presented in Supplementary Figures 3, 4.

### 3.5 Network meta-analysis

#### 3.5.1 Network geometry

**Figure 2:**
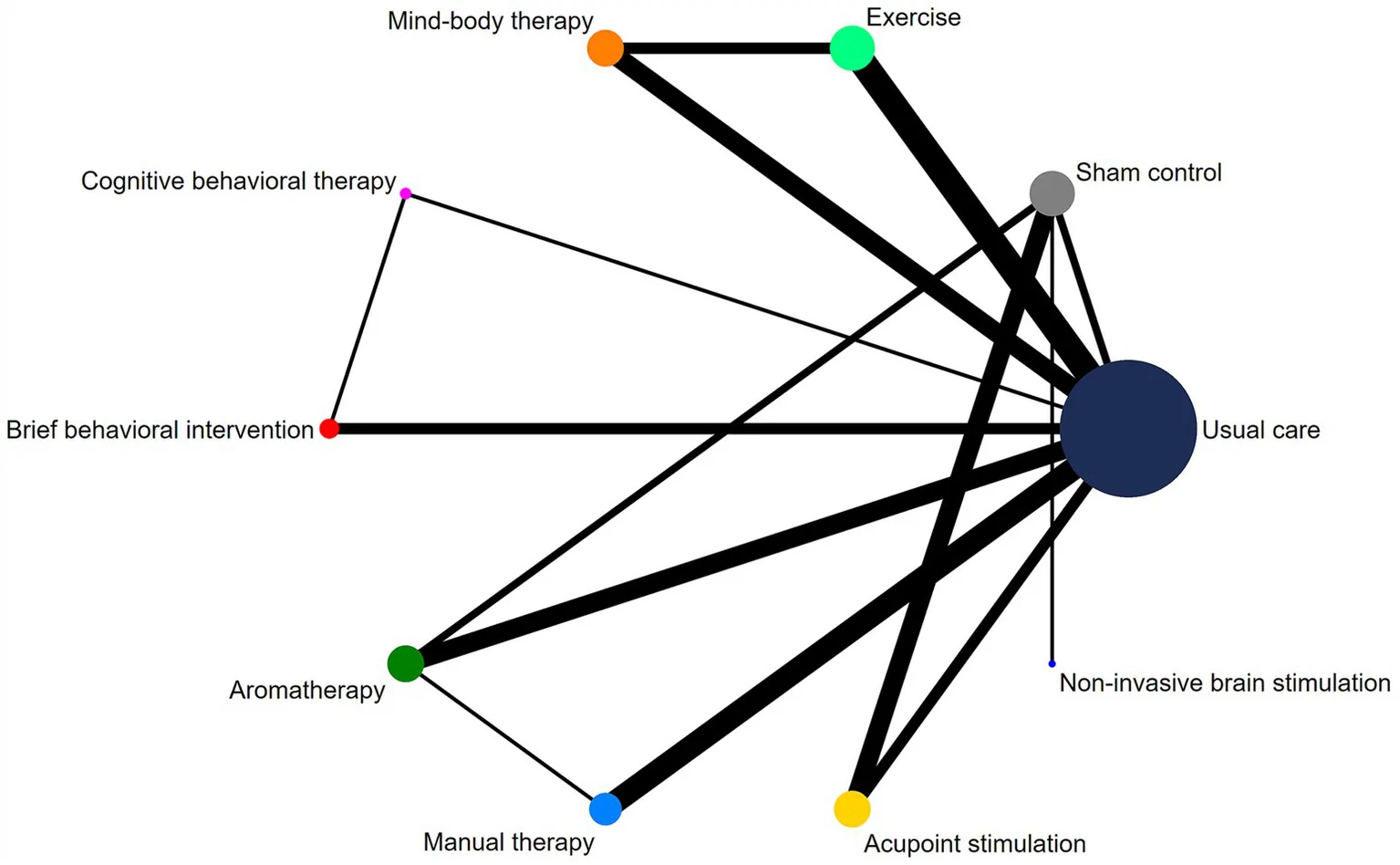
Network evidence plot for fatigue. Node sizes are proportional to the number of trials involving each node, and edge thicknesses are proportional to the number of trials contributing to each direct comparison.

The connected network comprised 10 nodes and 14 direct comparison edges (Figure 2). Usual care was the most highly connected node, and several closed loops allowed direct and indirect evidence to be compared. Evidence was sparse for CBT and non-invasive brain stimulation; the latter was represented by a single sham-controlled transcranial direct-current stimulation trial and had no direct comparison with usual care.

#### 3.5.2 Relative efficacy of interventions

**Figure 3:**
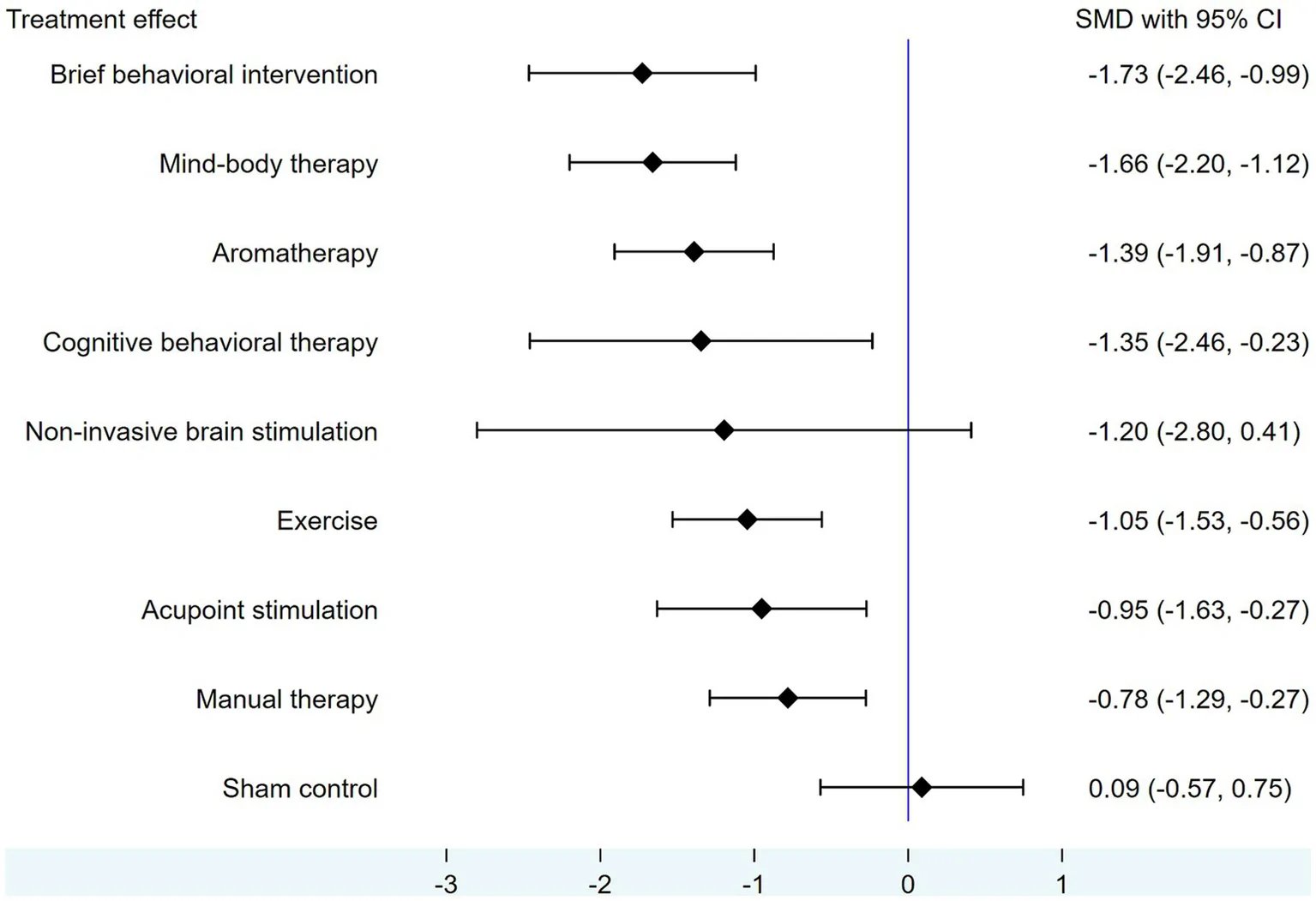
Network estimates versus usual care. Negative SMDs favor the intervention, and the vertical line indicates no effect.

Network estimates relative to usual care are shown in Figure 3, and all network comparisons are presented in Table 2. For comparisons with usual care, negative SMDs indicated lower fatigue scores than usual care.

**Table 2:** League table of network meta-analysis estimates for fatigue.

Brief behavioral intervention	Mind–body therapy	Aromatherapy	CBT	Non-invasive brain stimulation	Exercise	Acupoint stimulation	Manual therapy	Usual care	Sham control
−0.07 (−0.98, 0.85)^I^	Mind–body therapy								
−0.34 (−1.24, 0.56)^I^	−0.27 (−1.02, 0.48)^I^	Aromatherapy							
−0.38 (−1.45, 0.69)^M^	−0.31 (−1.55, 0.92)^I^	−0.05 (−1.27, 1.18)^I^	CBT						
−0.53 (−2.30, 1.23)^I^	−0.46 (−2.16, 1.23)^I^	−0.20 (−1.82, 1.43)^I^	−0.15 (−2.10, 1.80)^I^	Non-invasive brain stimulation					
−0.68 (−1.56, 0.20)^I^	**−0.61 (−1.21, −0.02)** ^**M**^	−0.35 (−1.05, 0.36)^I^	−0.30 (−1.51, 0.91)^I^	−0.15 (−1.83, 1.53)^I^	Exercise				
−0.78 (−1.78, 0.23)^I^	−0.71 (−1.58, 0.16)^I^	−0.44 (−1.21, 0.33)^I^	−0.39 (−1.70, 0.91)^I^	−0.24 (−1.82, 1.33)^I^	−0.09 (−0.93, 0.74)^I^	Acupoint stimulation			
**−0.95 (−1.84, −0.05)** ^**I**^	**−0.88 (−1.62, −0.14)** ^**I**^	−0.61 (−1.28, 0.06)^M^	−0.56 (−1.79, 0.66)^I^	−0.41 (−2.09, 1.26)^I^	−0.26 (−0.97, 0.44)^I^	−0.17 (−1.01, 0.67)^I^	Manual therapy		
**−1.73 (−2.46, −0.99)** ^**M**^	**−1.66 (−2.20, −1.12)** ^**M**^	**−1.39 (−1.91, −0.87)** ^**M**^	**−1.35 (−2.46, −0.23)** ^**M**^	−1.20 (−2.80, 0.41)^I^	**−1.05 (−1.53, −0.56)** ^**M**^	**−0.95 (−1.63, −0.27)** ^**M**^	**−0.78 (−1.29, −0.27)** ^**M**^	Usual care	
**−1.82 (−2.80, −0.83)** ^**I**^	**−1.75 (−2.60, −0.90)** ^**I**^	**−1.48 (−2.18, −0.78)** ^**M**^	**−1.43 (−2.73, −0.14)** ^**I**^	−1.28 (−2.75, 0.18)^D^	**−1.13 (−1.95, −0.32)** ^**I**^	**−1.04 (−1.61, −0.47)** ^**M**^	**−0.87 (−1.68, −0.06)** ^**I**^	−0.09 (−0.75, 0.57)^M^	Sham control

Treatments are ordered according to the primary SUCRA ranking. Values are SMDs with 95% CIs. Estimates compare the treatment in the column with the treatment in the row; negative values favor the column treatment. Bold values indicate estimates with 95% CIs that exclude zero. ^M^, mixed direct and indirect evidence; ^I^, indirect evidence only; ^D^, direct evidence only. CI, confidence interval; SMD, standardized mean difference; SUCRA, surface under the cumulative ranking curve. Yellow diagonal cells indicate treatment names; light green cells indicate 95% CIs including zero; darker green cells indicate 95% CIs excluding zero.

Compared with usual care, the most favorable estimates were observed for brief behavioral intervention (SMD = −1.73, 95% CI − 2.46 to −0.99), mind–body therapy (SMD = −1.66, 95% CI − 2.20 to −1.12), and aromatherapy (SMD = −1.39, 95% CI − 1.91 to −0.87). Lower fatigue scores were also observed with CBT (SMD = −1.35, 95% CI − 2.46 to −0.23), exercise (SMD = −1.05, 95% CI − 1.53 to −0.56), acupoint stimulation (SMD = −0.95, 95% CI − 1.63 to −0.27), and manual therapy (SMD = −0.78, 95% CI − 1.29 to −0.27).

The estimates for sham control (SMD = 0.09, 95% CI − 0.57 to 0.75) and non-invasive brain stimulation (SMD = −1.20, 95% CI − 2.80 to 0.41) were inconclusive; the non-invasive brain stimulation estimate was based on indirect evidence only. Between-study heterogeneity was substantial (*τ* = 0.642; τ^2^ = 0.412).

#### 3.5.3 Treatment ranking

**Figure 4:**
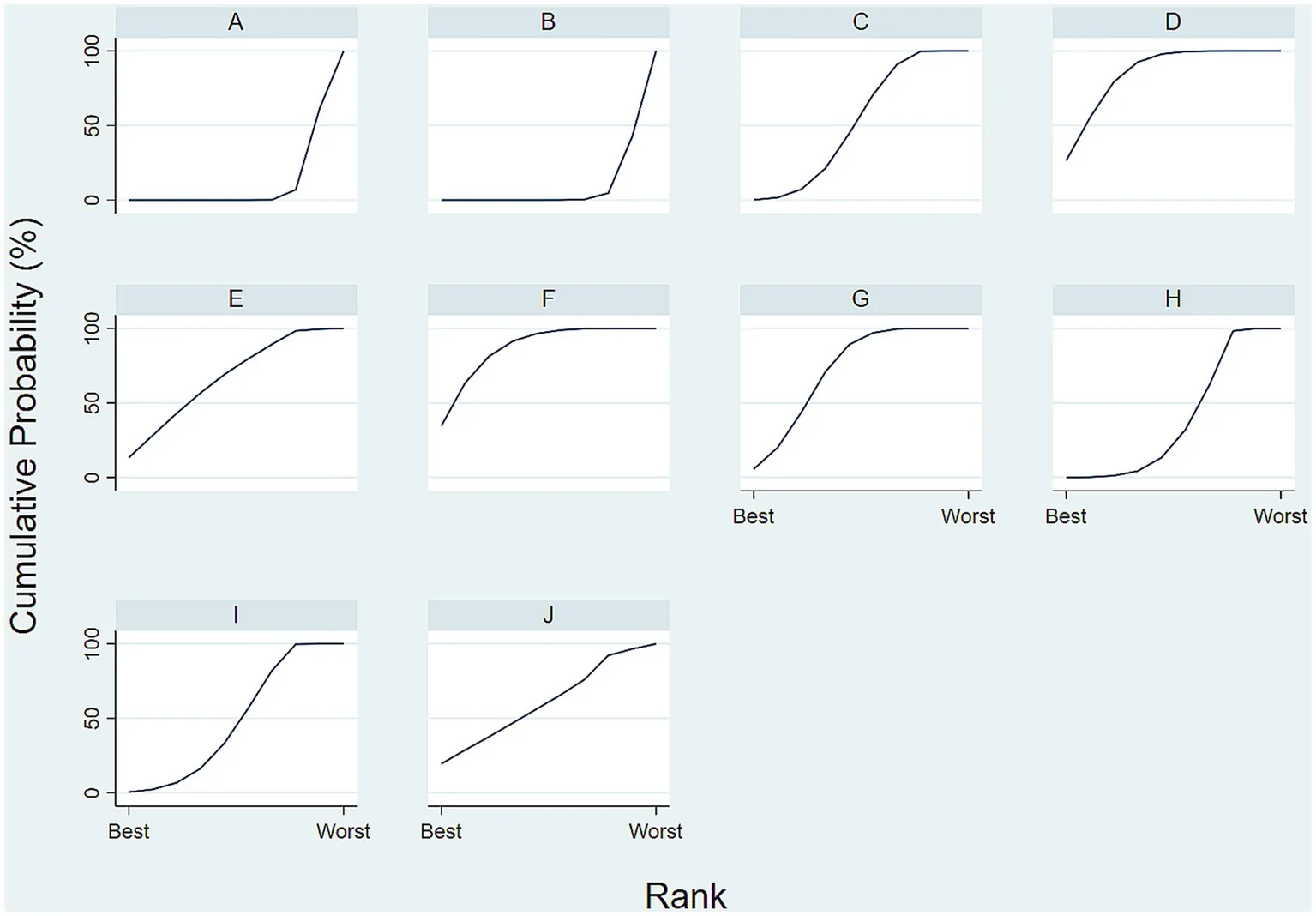
SUCRA ranking profiles. Panels **(A–J)** represent usual care, sham control, exercise, mind–body therapy, CBT, brief behavioral intervention, aromatherapy, manual therapy, acupoint stimulation, and non-invasive brain stimulation, respectively.

The SUCRA ranking profiles are presented in Figure 4. Brief behavioral intervention and mind–body therapy had the most favorable rank profiles. However, the network estimate comparing these two interventions was inconclusive (SMD = −0.07, 95% CI − 0.98 to 0.85), and most comparisons among the higher-ranked interventions also had 95% CIs crossing zero (Table 2). The ranking results should therefore be interpreted cautiously.

### 3.6 Inconsistency testing

The design-by-treatment interaction model found no evidence of global inconsistency (χ^2^ = 6.38, df = 8, *p* = 0.605). Similarly, none of the node-splitting analyses identified a statistically significant difference between direct and indirect estimates (all *p* > 0.05).

The sham control versus usual care comparison showed the largest direct–indirect discrepancy, but the corresponding CIs were wide and overlapping, and the node-splitting result did not reach statistical significance. Detailed inconsistency results are presented in Supplementary Table 6.

### 3.7 Small-study effects and publication bias

The comparison-adjusted funnel plot appeared asymmetric, suggesting possible small-study effects or publication bias. However, because no direct comparison included at least 10 trials, formal testing for asymmetry was not performed and the plot should be interpreted cautiously (Supplementary Figure 5).

### 3.8 Network meta-regression

Exploratory univariable network meta-regression analyses found no statistically significant effect modification by intervention duration (*p* = 0.366), geographic region (Iran or Türkiye versus other regions; *p* = 0.934), mean participant age (*p* = 0.751), mean dialysis vintage (*p* = 0.279), publication year analyzed continuously (*p* = 0.703), or publication period (>2021 versus ≤2021; *p* = 0.844).

In contrast, fatigue scale type was associated with differences in relative treatment effects (global χ^2^ = 34.60, df = 18, *p* = 0.011). Scale use was uneven: FSS was used most often in aromatherapy and manual-therapy trials, PFS in mind–body and acupoint-stimulation trials, and all four MFI trials were conducted in Iran or Türkiye, three after 2021 (Supplementary Table 3). No scale was consistently associated with larger or smaller effects across comparisons. The FSS/PFS-restricted network was disconnected, but effect directions were generally similar for the retained active interventions in the main connected component. These exploratory findings should be interpreted cautiously. Full meta-regression results are presented in Supplementary Table 7, and the scale distributions and restricted analysis in Supplementary Table 3.

### 3.9 Sensitivity analyses

After excluding the 10 trials judged to have a high overall risk of bias, 29 trials and all 10 nodes remained. The direction and statistical significance of the seven active-intervention estimates were maintained, whereas sham control and non-invasive brain stimulation remained inconclusive. The estimates for brief behavioral intervention, mind–body therapy, and aromatherapy were −1.69 (95% CI − 2.62 to −0.76), −1.65 (95% CI − 2.17 to −1.13), and −1.15 (95% CI − 1.81 to −0.49), respectively (Table 3).

**Table 3:** Primary and sensitivity network estimates versus usual care.

Intervention	Primary analysis SMD (95% CI)	Excluding high-risk trials SMD (95% CI)	Excluding trials with any analytical arm n < 20 SMD (95% CI)	Excluding multicomponent- intervention trials SMD (95% CI)
Brief behavioral intervention	−1.73 (−2.46 to −0.99)	−1.69 (−2.62 to −0.76)	−1.92 (−2.72 to −1.12)	−1.92 (−2.72 to −1.12)
Mind–body therapy	−1.66 (−2.20 to −1.12)	−1.65 (−2.17 to −1.13)	−1.59 (−2.18 to −1.01)	−1.45 (−2.04 to −0.86)
Aromatherapy	−1.39 (−1.91 to −0.87)	−1.15 (−1.81 to −0.49)	−1.39 (−1.92 to −0.87)	−1.34 (−1.96 to −0.72)
CBT	−1.35 (−2.46 to −0.23)	−1.87 (−3.46 to −0.28)	−2.10 (−3.68 to −0.52)	−0.56 (−2.17 to 1.05)
Non-invasive brain stimulation	−1.20 (−2.80 to 0.41)	−1.42 (−3.04 to 0.19)	Not estimable	−1.20 (−2.85 to 0.45)
Exercise	−1.05 (−1.53 to −0.56)	−1.03 (−1.52 to −0.53)	−0.84 (−1.46 to −0.21)	−0.98 (−1.48 to −0.49)
Acupoint stimulation	−0.95 (−1.63 to −0.27)	−1.24 (−1.96 to −0.52)	−0.95 (−1.64 to −0.26)	−0.98 (−1.79 to −0.18)
Manual therapy	−0.78 (−1.29 to −0.27)	−0.85 (−1.44 to −0.26)	−0.78 (−1.30 to −0.27)	−0.95 (−1.63 to −0.27)
Sham control	0.09 (−0.57 to 0.75)	−0.14 (−0.93 to 0.65)	0.09 (−0.58 to 0.76)	0.08 (−0.64 to 0.81)

Negative SMDs favor the intervention. Not estimable indicates that the corresponding node was absent from the restricted network. CBT, cognitive behavioral therapy; CI, confidence interval; SMD, standardized mean difference.

Excluding six trials in which at least one analytical arm included fewer than 20 participants retained 33 trials and nine nodes. Non-invasive brain stimulation was removed because its only contributing trial met the small-arm criterion. Most estimates remained directionally consistent with the primary analysis; the exercise estimate was attenuated to −0.84 (95% CI − 1.46 to −0.21), whereas the CBT estimate became more extreme at −2.10 (95% CI − 3.68 to −0.52) but remained based on sparse evidence (Table 3).

Excluding the seven trials containing multicomponent interventions retained 32 trials, 67 analytical arms, all 10 nodes, and 2,231 participants, and the network remained connected. Most estimates remained directionally similar to the primary analysis; however, the CBT estimate attenuated to −0.56 (95% CI − 2.17 to 1.05) and became inconclusive (Table 3).

### 3.10 Confidence in the evidence

For comparisons with usual care, confidence in the evidence was moderate for mind–body therapy, brief behavioral intervention, and aromatherapy; low for exercise, manual therapy, acupoint stimulation, and sham control; and very low for CBT and non-invasive brain stimulation.

**Figure 5:**
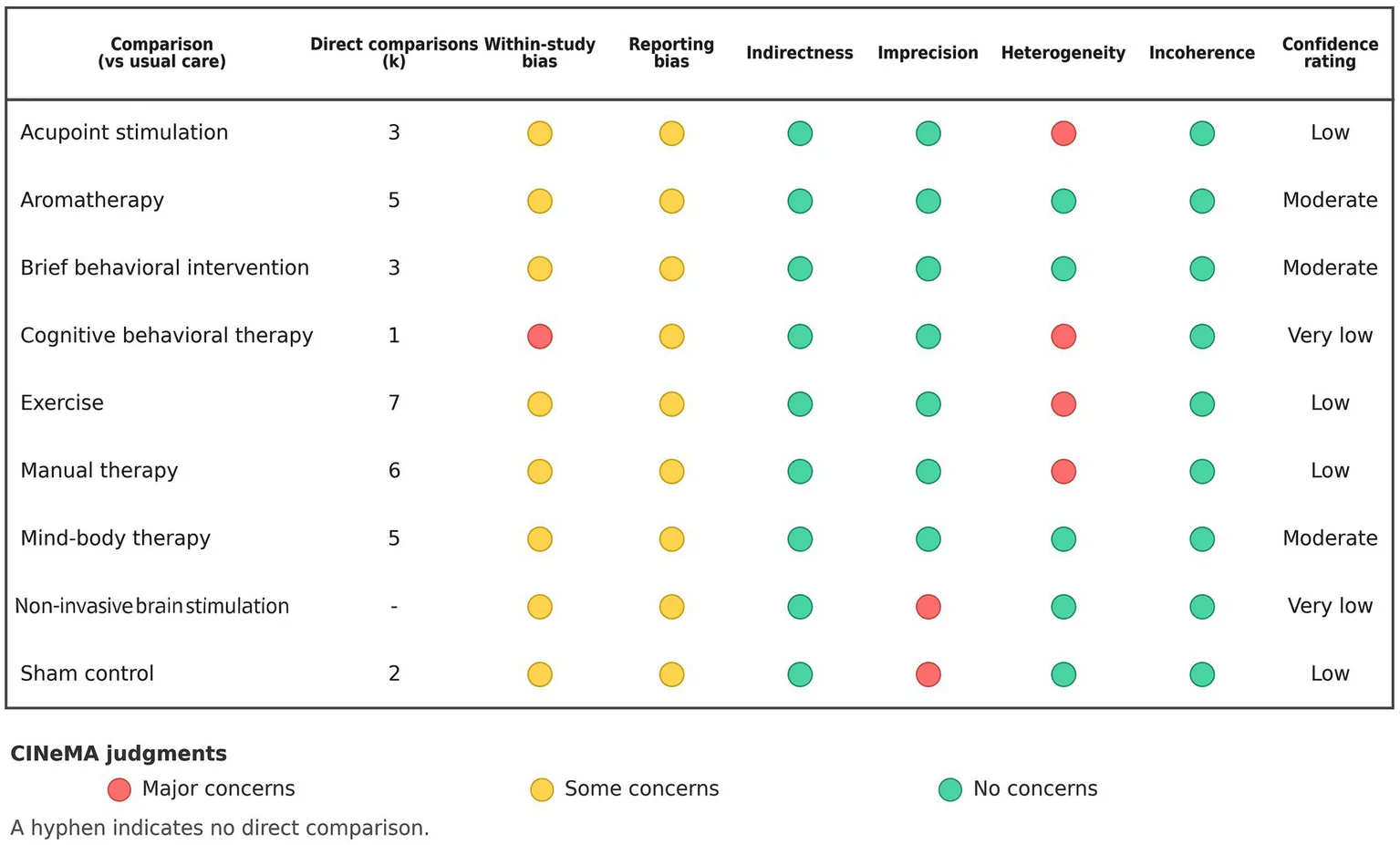
Confidence in the evidence for network estimates versus usual care.

Downgrading mainly reflected within-study bias, suspected reporting bias, heterogeneity, and imprecision. Evidence was especially sparse for CBT and non-invasive brain stimulation, further limiting the interpretability of these estimates. No comparison was rated as high confidence. The CINeMA judgments for comparisons with usual care are summarized in Figure 5, whereas assessments for all 45 network comparisons are provided in Supplementary Table 8.

## 4 Discussion

### 4.1 Summary and interpretation of findings

This NMA included 39 RCTs involving 2,943 participants and compared eight active non-pharmacological interventions, sham control, and usual care for fatigue in people receiving MHD. Brief behavioral intervention and mind–body therapy showed the most favorable estimates and ranking profiles, followed by aromatherapy. CBT, exercise, acupoint stimulation, and manual therapy were also associated with lower fatigue scores than usual care, whereas the estimates for non-invasive brain stimulation and sham control were inconclusive. However, brief behavioral intervention and mind–body therapy did not differ clearly from each other, and most comparisons between active interventions were imprecise. The findings therefore identify several promising intervention categories but do not establish a single definitively superior approach.

These findings are broadly consistent with previous evidence syntheses. The latest Cochrane review concluded that exercise, aromatherapy, massage, and acupressure may reduce fatigue in people receiving dialysis, although the evidence was generally limited in certainty and precision (17). Recent reviews have similarly reported potential benefits of social-psychological interventions (44), intradialytic exercise (45), acupressure (46), and aromatherapy (28). The present study extends this evidence by comparing a broader range of intervention categories within a common network, integrating direct and indirect evidence, and evaluating confidence in each network estimate. Nevertheless, the network included variation in participant characteristics, intervention protocols, fatigue measures, and clinical settings. The results should therefore be interpreted as a comparative synthesis of heterogeneous evidence rather than as a fixed treatment hierarchy.

A notable finding was that brief behavioral intervention and mind–body therapy had the most favorable estimates relative to usual care. The brief behavioral intervention node included energy-conservation education, recreational therapy, humor therapy, and other structured fatigue-management or psychoeducational strategies (19, 20, 44). Mind–body therapy included relaxation-based approaches, yoga-related exercise, Reiki, and interventions combining mental and bodily regulation (25, 47). Although the components differed, both categories sought to improve how individuals regulate activity, cope with symptoms, and respond to the physical and psychological demands of hemodialysis (48).

This pattern is clinically plausible given the multidimensional nature of dialysis-related fatigue (49, 50). Fatigue in kidney failure may reflect interacting physical, psychological, cognitive, and treatment-related factors, including deconditioning, sleep disturbance, inflammation, psychological distress, and reduced perceived control (8, 51–53). Behavioral interventions may support pacing, energy conservation, coping, and self-efficacy, whereas mind–body approaches may promote relaxation, emotional regulation, and gradual physical engagement (30, 53). Evidence from broader fatigue and stress literature also suggests that movement-based mind–body practices may influence perceived fatigue and stress-related physiological regulation (54, 55). These mechanisms were not directly tested in the included trials and should be regarded as possible explanations rather than confirmed mediating pathways.

Estimates for brief behavioral intervention and mind–body therapy remained directionally similar across the three sensitivity analyses. However, the CBT estimate attenuated and became inconclusive after multicomponent trials were excluded, indicating sensitivity to individual studies and classification decisions. These findings indicate that the direction of several estimates was maintained, but they do not establish robustness; effect-size inflation and instability remain possible. Only four trials contributed to the brief behavioral intervention node, intervention content varied within both categories, and many comparisons with other active interventions relied partly or entirely on indirect evidence. In addition, the network comparison between brief behavioral intervention and mind–body therapy was inconclusive. Their favorable rankings should therefore be considered hypothesis-generating and should not be used to infer that one intervention component or delivery format is optimal.

Aromatherapy also showed a relatively favorable estimate, with moderate-confidence evidence compared with usual care. Its observed benefit may relate to relaxation, emotional responses, or sleep (23, 56, 57), although the present analysis could not distinguish effects attributable to the essential oils from expectation, attention, treatment ritual, or other nonspecific components. Exercise, manual therapy, and acupoint stimulation were associated with lower fatigue scores, but confidence was low because of study limitations, heterogeneity, and suspected reporting bias.

The position of exercise below brief behavioral intervention and mind–body therapy in the ranking should not be interpreted as evidence of lower overall clinical value. Exercise may also improve physical capacity, muscle strength, mobility, and other outcomes that were not evaluated in this fatigue network (18, 58–60). Its clinical relevance may therefore be greater for individuals whose fatigue is accompanied by deconditioning or functional limitation (61, 62). Similarly, manual therapy and acupoint stimulation may be appropriate for individuals who prefer complementary or less physically demanding approaches. The rankings represent average comparative estimates for fatigue and do not define a universal sequence of clinical treatment.

The CBT and non-invasive brain stimulation findings illustrate the distinction between statistical significance and confidence in the evidence. CBT was associated with lower fatigue scores than usual care, but only two trials contributed to the node, confidence was very low, and the CI was wide. Its estimate became more extreme after trials with small analytical arms were excluded, indicating instability rather than increased certainty. Non-invasive brain stimulation was represented by one small sham-controlled transcranial direct-current stimulation trial, and its indirect estimate versus usual care crossed the null. Neither intervention should therefore be prioritized clinically on the basis of its point estimate or ranking alone.

The findings for sham control also require caution. Sham procedures differed across intervention classes and included placebo aromatherapy, sham acupoint stimulation, and sham transcranial direct-current stimulation. Although the direct pairwise estimate favored sham control over usual care, the network estimate was inconclusive. This does not demonstrate equivalence between sham control and usual care and cannot establish the absence of expectation, attention, placebo, or other nonspecific effects (63). The direct–indirect discrepancy was not statistically significant, but the wide CIs indicate substantial uncertainty.

No statistically significant global or local inconsistency was detected, suggesting no clear statistical disagreement between the direct and indirect evidence. However, non-significant inconsistency tests do not confirm that transitivity was fully satisfied (38, 40). Interventions were classified according to their predominant therapeutic components, with acupoint stimulation separated from non-invasive brain stimulation and no generic combined-therapy node used. Residual variation nevertheless remained in intervention content, intensity, duration, delivery, and practitioner involvement. Geographic concentration, incomplete reporting of dialysis vintage, sparse evidence for several nodes, and uneven distribution of fatigue instruments further limited the transitivity assessment.

The significant scale-type association may reflect differences in the fatigue constructs measured (64), but also structural confounding because scale use was uneven across intervention nodes and study settings. Although effect directions were generally similar in the main connected FSS/PFS component, the restricted network was disconnected and did not permit CBT or brief behavioral intervention to be compared with usual care. The scale-type finding should therefore be regarded as exploratory rather than attributed solely to measurement properties.

Although the FSS and PFS were the most frequently used instruments, back-translating SMDs would require selecting a representative standard deviation from heterogeneous trials and could imply unsupported comparability across instruments and populations. A common scale-specific minimum clinically important difference was therefore not applied (64–66). The CINeMA equivalence range of −0.20 to 0.20 SMD units was an analytical threshold rather than a validated patient-perceived threshold. Accordingly, large SMDs should not automatically be interpreted as equally large clinical benefits.

Overall, confidence was moderate for brief behavioral intervention, mind–body therapy, and aromatherapy compared with usual care; low for exercise, manual therapy, acupoint stimulation, and sham control; and very low for CBT and non-invasive brain stimulation. SUCRA rankings should therefore be regarded as descriptive summaries of ranking probabilities and interpreted together with effect estimates, CIs, evidence sources, heterogeneity, and CINeMA judgments (39, 43).

### 4.2 Strengths and limitations of this study

This review included only RCTs and combined conventional pairwise meta-analysis with NMA, allowing direct evidence to be distinguished from network estimates. Intervention nodes were defined according to their predominant therapeutic components to improve clinical interpretability. Although study-level classification decisions were documented and examined in a sensitivity analysis, assigning complex intervention packages to a single predominant component may still oversimplify interactions between individual treatment components. Global and local inconsistency were assessed separately, potential effect modifiers were explored through network meta-regression, and sensitivity analyses examined the influence of high overall risk of bias, small analytical arms, and multicomponent classification decisions. Confidence in each network estimate was systematically evaluated using CINeMA.

Several limitations should be acknowledged. Fatigue was not always the primary outcome, and some trials reported multiple fatigue measures or assessment times, creating potential for selective outcome reporting (67). Only two trials were judged to have a low overall risk of bias, whereas 27 raised some concerns and 10 were at high risk. Fatigue was predominantly measured using self-reported instruments, and participant blinding was generally infeasible because of the nature of the interventions. These features increase susceptibility to expectation effects and bias in outcome measurement (36, 68). Although exclusion of high-risk trials broadly preserved the direction and statistical significance of the primary estimates, inflation of effect magnitude cannot be excluded.

Substantial heterogeneity was observed in many direct comparisons and in the overall network. Fatigue scale type was the only statistically significant effect modifier identified, but scale use was uneven across nodes and regions, and the FSS/PFS restriction produced a disconnected network, limiting causal interpretation. Other potentially important modifiers, including baseline fatigue severity, intervention intensity, depression, sleep disturbance, comorbidities, adherence, and co-interventions, were inconsistently reported and could not be examined reliably (52, 69, 70).

The network was sparse for several interventions, and many active-treatment comparisons relied predominantly or entirely on indirect evidence. The comparison-adjusted funnel plot suggested possible small-study effects or publication bias, but formal asymmetry testing was not appropriate because no direct comparison included at least 10 trials (42). The attenuation of the exercise estimate and the more extreme CBT estimate after excluding trials with small analytical arms further indicate that some findings remain sensitive to sparse evidence.

The analysis primarily reflected outcomes measured at completion of the planned intervention. Inconsistent reporting of later follow-up prevented reliable evaluation of whether benefits persisted after treatment ended. In addition, 27 of the 39 trials were conducted in Iran or Türkiye. Although geographic region was not identified as a statistically significant effect modifier, the analysis had limited power to detect regional differences, and generalizability to underrepresented healthcare systems remains uncertain.

Finally, dialysis vintage was available in only 23 trials, and dialysis adequacy measures such as Kt/V were inconsistently reported. These factors may influence fatigue severity and treatment response but could not be evaluated comprehensively (13). Restriction to English-language publications may also have introduced language bias.

### 4.3 Implications for clinical practice and future research

The findings support consideration of several non-pharmacological approaches for fatigue management, but treatment selection should not be based on SUCRA rankings alone. Brief behavioral intervention, mind–body therapy, and aromatherapy appear promising because they showed relatively favorable estimates and moderate-confidence evidence compared with usual care. Exercise, manual therapy, and acupoint stimulation may also be considered as adjunctive options, although confidence in their effects was low (45, 46, 57). Evidence for CBT and non-invasive brain stimulation remains insufficient to support their routine prioritization.

Clinical decisions should consider the characteristics and potential contributors to fatigue, physical capacity, comorbidities, treatment burden, patient preferences, cultural acceptability, safety, staff expertise, and available resources (71, 72). Nephrology nurses are well positioned to assess fatigue, identify modifiable contributing factors, support shared decision-making, coordinate intervention delivery, and monitor adherence and response (26, 60, 73). The present findings should inform individualized symptom management rather than replace clinical judgment.

Future research should prioritize adequately powered, multicenter trials that directly compare active interventions rather than relying predominantly on usual care controls. For behavioral and educational interventions, digital or hybrid delivery models may also be evaluated as scalable approaches to intervention delivery, symptom monitoring, and follow-up (74). Intervention protocols should clearly report content, intensity, frequency, duration, provider training, adherence, adverse events, and co-interventions (75). Fatigue outcomes should be prespecified and measured using psychometrically supported instruments with clinically interpretable scoring (66, 76), and both end-of-intervention and longer-term follow-up outcomes should be reported.

Future trials should also report baseline fatigue severity, dialysis vintage, dialysis adequacy, psychological distress, sleep disturbance, comorbidities, and concomitant treatments. Greater geographic diversity and more direct comparisons between active interventions would improve the transferability of findings and strengthen the evidence network.

## 5 Conclusion

This systematic review and NMA suggests that several non-pharmacological interventions may reduce fatigue in people receiving MHD. Brief behavioral intervention and mind–body therapy appeared to be the most promising options based on their effect estimates and ranking profiles, while aromatherapy was also supported by moderate-confidence evidence. However, limited head-to-head comparisons, substantial heterogeneity, possible small-study effects, and confidence ranging from very low to moderate do not support a definitive treatment hierarchy. Intervention selection should therefore be individualized according to fatigue characteristics, patient preferences, safety, feasibility, staff expertise, and available resources. Adequately powered multicenter trials using active comparators, standardized fatigue measures, and longer-term follow-up are needed.

## Data Availability

The original contributions presented in the study are included in the article/Supplementary material, further inquiries can be directed to the corresponding author/s.
