# Supplementary material for: Comparative efficacy of non-pharmacological interventions on fatigue in people undergoing hemodialysis: a systematic review and network meta-analysis
Source: Front Med (Lausanne). 2026 Aug 19;13:1862895. doi: 10.3389/fmed.2026.1862895 (PMC13533668; doi:10.3389/fmed.2026.1862895)
Supplement: Supplementary file 1 [file Table_1.docx]

Supplementary Table 1. Search strategies

| **Database** | **Search Strategy** | **Articles Retrieved** |
| --- | --- | --- |
| **PubMed** | #1: "Renal Dialysis"[MeSH Terms] OR "Hemodiafiltration"[MeSH Terms] OR "Renal Replacement Therapy"[MeSH Terms]  #2: "hemodialy*"[Title/Abstract] OR "haemodialy*"[Title/Abstract] OR "Hemodiafiltration"[Title/Abstract] OR "hemofiltration"[Title/Abstract] OR "haemofiltration"[Title/Abstract] OR "dialysis"[Title/Abstract] OR "dialyses"[Title/Abstract] OR "blood purification"[Title/Abstract] OR "maintenance hemodialysis"[Title/Abstract]  #3: #1 OR #2  #4: "Complementary Therapies"[MeSH Terms]  #5: "nonpharmacologic*"[Title/Abstract] OR "non pharmacologic*"[Title/Abstract] OR "non drug*"[Title/Abstract] OR "nondrug*"[Title/Abstract]  #6: "Acupressure"[MeSH Terms]  #7: "Acupressure"[Title/Abstract] OR "acupoint*"[Title/Abstract] OR "auricular point"[Title/Abstract] OR "ear point"[Title/Abstract] OR "shiatsu"[Title/Abstract]  #8: "Acupuncture Therapy"[MeSH Terms]  #9: "acupuncture"[Title/Abstract] OR "electroacupuncture"[Title/Abstract] OR "needle therap*"[Title/Abstract]  #10: "Aromatherapy"[MeSH Terms]  #11: "aromatherap*"[Title/Abstract] OR "aroma therap*"[Title/Abstract] OR "lavender"[Title/Abstract] OR "essential oil*"[Title/Abstract] OR "lavender oil"[Title/Abstract]  #12: "Massage"[MeSH Terms]  #13: "Massage"[Title/Abstract] OR "tuina"[Title/Abstract] OR "manual therap*"[Title/Abstract] OR "rubbing"[Title/Abstract] OR "effleurage"[Title/Abstract]  #14: "reflexology"[Title/Abstract] OR "foot massage"[Title/Abstract] OR "foot zone"[Title/Abstract] OR "zone therapy"[Title/Abstract] OR "reflexotherap*"[Title/Abstract]  #15: "Exercise"[MeSH Terms] OR "Exercise Therapy"[MeSH Terms] OR "Resistance Training"[MeSH Terms] OR "Yoga"[MeSH Terms] OR "Tai Ji"[MeSH Terms] OR "Walking"[MeSH Terms] OR "Qigong"[MeSH Terms]  #16: "exercis*"[Title/Abstract] OR "aerobic training"[Title/Abstract] OR "Resistance Training"[Title/Abstract] OR "strength training"[Title/Abstract] OR "weight training"[Title/Abstract] OR "physical training"[Title/Abstract] OR "physical activit*"[Title/Abstract] OR "intradialytic training"[Title/Abstract] OR "walk*"[Title/Abstract] OR "cycl*"[Title/Abstract] OR "bicycle"[Title/Abstract] OR "treadmill"[Title/Abstract] OR "ergometer"[Title/Abstract] OR "Yoga"[Title/Abstract] OR "pilates"[Title/Abstract] OR "tai chi"[Title/Abstract] OR "Qigong"[Title/Abstract] OR "baduanjin"[Title/Abstract]  #17: "Mind-Body Therapies"[MeSH Terms] OR "Mindfulness"[MeSH Terms] OR "Meditation"[MeSH Terms] OR "Relaxation Therapy"[MeSH Terms]  #18: "mind-body"[Title/Abstract] OR "mindful*"[Title/Abstract] OR "meditati*"[Title/Abstract] OR "relaxation"[Title/Abstract]  #19: "Psychotherapy"[MeSH Terms] OR "Behavior Therapy"[MeSH Terms] OR "Cognitive Behavioral Therapy"[MeSH Terms]  #20: ("psycholog*"[Title/Abstract] AND ("therap*"[Title/Abstract] OR "intervention*"[Title/Abstract] OR "treatment*"[Title/Abstract] OR "counsel*"[Title/Abstract])) OR "behavioral intervention*"[Title/Abstract] OR "behavioural intervention*"[Title/Abstract] OR "cognitive behavioral therap*"[Title/Abstract] OR "cognitive behavioural therap*"[Title/Abstract] OR "cognitive behavioral treatment*"[Title/Abstract] OR "cognitive behavioural treatment*"[Title/Abstract] OR "CBT"[Title/Abstract]  #21: "Music Therapy"[MeSH Terms]  #22: "Music*"[Title/Abstract]  #23: "Health Education"[MeSH Terms] OR "Patient Education as Topic"[MeSH Terms]  #24: "education program*"[Title/Abstract] OR "patient education"[Title/Abstract] OR "Health Education"[Title/Abstract] OR "education intervention*"[Title/Abstract] OR "educational intervention*"[Title/Abstract] OR "self management"[Title/Abstract] OR "self care"[Title/Abstract]  #25: "Telemedicine"[MeSH Terms] OR "Remote Consultation"[MeSH Terms] OR "Telerehabilitation"[MeSH Terms] OR "Internet"[MeSH Terms] OR "Internet-Based Intervention"[MeSH Terms] OR "Therapy, computer assisted"[MeSH Terms] OR "Mobile Applications"[MeSH Terms] OR "Smartphone"[MeSH Terms] OR "Cell Phone"[MeSH Terms] OR "Text Messaging"[MeSH Terms] OR "Wearable Electronic Devices"[MeSH Terms] OR "Fitness Trackers"[MeSH Terms] OR "Wireless Technology"[MeSH Terms] OR "Virtual Reality"[MeSH Terms] OR "Exergaming"[MeSH Terms] OR "Video Games"[MeSH Terms] OR "Robotics"[MeSH Terms] OR "Artificial Intelligence"[MeSH Terms]  #26: "telemedicine"[Title/Abstract] OR "telehealth*"[Title/Abstract] OR "telecare"[Title/Abstract] OR "ehealth"[Title/Abstract] OR "mhealth"[Title/Abstract] OR "digital health"[Title/Abstract] OR "remote consult*"[Title/Abstract] OR "video consult*"[Title/Abstract] OR "teleconsult*"[Title/Abstract] OR "virtual visit*"[Title/Abstract] OR "video visit*"[Title/Abstract] OR ("mobile"[Title/Abstract] AND ("app"[Title/Abstract] OR "apps"[Title/Abstract] OR "application*"[Title/Abstract])) OR "smartphone*"[Title/Abstract] OR "internet-based"[Title/Abstract] OR "web-based"[Title/Abstract] OR "online intervention*"[Title/Abstract] OR "online program*"[Title/Abstract] OR "wearable*"[Title/Abstract] OR "pedometer*"[Title/Abstract] OR "accelerometer*"[Title/Abstract] OR "virtual reality"[Title/Abstract] OR "exergame*"[Title/Abstract] OR "video game*"[Title/Abstract] OR "gamification"[Title/Abstract] OR "robot*"[Title/Abstract] OR "artificial intelligence"[Title/Abstract]  #27: #4 OR #5 OR #6 OR #7 OR #8 OR #9 OR #10 OR #11 OR #12 OR #13 OR #14 OR #15 OR #16 OR #17 OR #18 OR #19 OR #20 OR #21 OR #22 OR #23 OR #24 OR #25 OR #26  #28: "Fatigue"[MeSH Terms]  #29: "fatigue*"[Title/Abstract] OR "tiredness"[Title/Abstract] OR "weary"[Title/Abstract] OR "weariness"[Title/Abstract] OR "exhaustion"[Title/Abstract] OR "exhausted"[Title/Abstract] OR "tired"[Title/Abstract] OR "vitality"[Title/Abstract] OR "lassitude"[Title/Abstract]  #30: #28 OR #29  #31: ("randomized controlled trial"[Publication Type] OR "controlled clinical trial"[Publication Type] OR "randomized"[Title/Abstract] OR "randomised"[Title/Abstract] OR "placebo"[Title/Abstract] OR "drug therapy"[MeSH Subheading] OR "randomly"[Title/Abstract] OR "trial"[Title/Abstract] OR "groups"[Title/Abstract]) NOT ("animals"[MeSH Terms] NOT "humans"[MeSH Terms])  #32: #3 AND #27 AND #30 AND #31 | 2025.12.14  Items found:  291 |
| **Embase** | #1: 'renal replacement therapy'/exp OR 'hemodialysis'/exp OR 'hemodiafiltration'/exp OR ('hemodialy*' OR 'haemodialy*' OR 'hemodiafiltration' OR 'hemofiltration' OR 'haemofiltration' OR 'dialysis' OR 'dialyses' OR 'blood purification' OR 'maintenance hemodialysis'):ti,ab,kw  #2: 'alternative medicine'/exp OR ('nonpharmacologic*' OR 'non pharmacologic*' OR 'non drug*' OR 'nondrug*'):ti,ab,kw  #3: 'acupressure'/exp OR ('acupressure' OR 'acupoint*' OR 'auricular point' OR 'ear point' OR 'shiatsu'):ti,ab,kw  #4: 'acupuncture'/exp OR ('acupuncture' OR 'electroacupuncture' OR 'needle therap*'):ti,ab,kw  #5: 'aromatherapy'/exp OR ('aromatherap*' OR 'aroma therap*' OR 'lavender' OR 'essential oil*' OR 'lavender oil'):ti,ab,kw  #6: 'massage'/exp OR ('massage' OR 'tuina' OR 'manual therap*' OR 'rubbing' OR 'effleurage'):ti,ab,kw  #7: 'reflexology'/exp OR ('reflexology' OR 'foot massage' OR 'foot zone' OR 'zone therapy' OR 'reflexotherap*'):ti,ab,kw  #8: 'exercise'/exp OR 'kinesiotherapy'/exp OR 'resistance training'/exp OR 'yoga'/exp OR 'tai chi'/exp OR 'walking'/exp OR 'qigong'/exp OR ('exercis*' OR 'aerobic training' OR 'resistance training' OR 'strength training' OR 'weight training' OR 'physical training' OR 'physical activit*' OR 'intradialytic training' OR 'walk*' OR 'cycl*' OR 'bicycle' OR 'treadmill' OR 'ergometer' OR 'yoga' OR 'pilates' OR 'tai chi' OR 'qigong' OR 'baduanjin'):ti,ab,kw  #9: 'mindfulness'/exp OR 'meditation'/exp OR 'relaxation training'/exp OR ('mind-body' OR 'mindful*' OR 'meditati*' OR 'relaxation'):ti,ab,kw  #10: 'psychotherapy'/exp OR 'behavior therapy'/exp OR 'cognitive behavioral therapy'/exp OR (('psycholog*' AND ('therap*' OR 'intervention*' OR 'treatment*' OR 'counsel*')) OR 'behavioral intervention*' OR 'behavioural intervention*' OR 'cognitive behavioral therap*' OR 'cognitive behavioural therap*' OR 'cognitive behavioral treatment*' OR 'cognitive behavioural treatment*' OR 'CBT'):ti,ab,kw  #11: 'music therapy'/exp OR 'music*':ti,ab,kw  #12: 'health education'/exp OR 'patient education'/exp OR ('education program*' OR 'patient education' OR 'health education' OR 'education intervention*' OR 'educational intervention*' OR 'self management' OR 'self care'):ti,ab,kw  #13: 'telemedicine'/exp OR 'teleconsultation'/exp OR 'telerehabilitation'/exp OR 'Internet'/exp OR 'web-based intervention'/exp OR 'computer assisted therapy'/exp OR 'mobile application'/exp OR 'smartphone'/exp OR 'mobile phone'/exp OR 'text messaging'/exp OR 'wearable computer'/exp OR 'activity monitor'/exp OR 'wireless communication'/exp OR 'virtual reality'/exp OR 'exergaming'/exp OR 'video game'/exp OR 'robotics'/exp OR 'artificial intelligence'/exp OR ('telemedicine' OR 'telehealth*' OR 'telecare' OR 'ehealth' OR 'mhealth' OR 'digital health' OR 'remote consult*' OR 'video consult*' OR 'teleconsult*' OR 'virtual visit*' OR 'video visit*' OR ('mobile' AND ('app' OR 'apps' OR 'application*')) OR 'smartphone*' OR 'internet-based' OR 'web-based' OR 'online intervention*' OR 'online program*' OR 'wearable*' OR 'pedometer*' OR 'accelerometer*' OR 'virtual reality' OR 'exergame*' OR 'video game*' OR 'gamification' OR 'robot*' OR 'artificial intelligence'):ti,ab,kw  #14: #2 OR #3 OR #4 OR #5 OR #6 OR #7 OR #8 OR #9 OR #10 OR #11 OR #12 OR #13  #15: 'fatigue'/exp OR ('fatigue*' OR 'tiredness' OR 'weary' OR 'weariness' OR 'exhaustion' OR 'exhausted' OR 'tired' OR 'vitality' OR 'lassitude'):ti,ab,kw  #16: 'randomized controlled trial'/exp OR 'controlled clinical trial'/de OR 'randomization'/de OR 'intermethod comparison'/de OR 'double blind procedure'/de OR 'human experiment'/de OR (random* OR placebo OR ('open' NEXT/1 'label') OR (('double' OR 'single' OR 'doubly' OR 'singly') NEXT/1 ('blind' OR 'blinded' OR 'blindly')) OR ('parallel' NEXT/1 'group*') OR (crossover OR 'cross over') OR (assigned OR allocated) OR (volunteer OR volunteers) OR ('controlled' NEAR/8 ('study' OR 'design' OR 'trial'))):ti,ab,tt OR (compare OR compared OR comparison OR trial):ti,tt OR ((evaluated OR evaluate OR evaluating OR assessed OR assess) AND (compare OR compared OR comparing OR comparison)):ab OR ((assign* OR match OR matched OR allocation) NEAR/6 (alternate OR group OR groups OR intervention OR interventions OR patient OR patients OR subject OR subjects OR participant OR participants)):ti,ab,tt  #17: (((random* NEXT/1 sampl* NEAR/8 ('cross section*' OR questionnaire* OR survey OR surveys OR database OR databases)):ti,ab,tt) NOT ('comparative study'/de OR 'controlled study'/de OR ('randomised controlled' OR 'randomized controlled' OR 'randomly assigned'):ti,ab,tt)) OR ('cross‐sectional study'/de NOT ('randomized controlled trial'/exp OR 'controlled clinical study'/de OR 'controlled study'/de OR ('randomised controlled' OR 'randomized controlled' OR 'control group' OR 'control groups'):ti,ab,tt)) OR (('case control*':ti,ab,tt AND random*:ti,ab,tt) NOT ('randomised controlled' OR 'randomized controlled'):ti,ab,tt) OR ('systematic review':ti,tt NOT (trial:ti,tt OR study:ti,tt)) OR (nonrandom*:ti,ab,tt NOT random*:ti,ab,tt) OR 'random field*':ti,ab,tt OR ('random cluster' NEAR/4 sampl*):ti,ab,tt OR ((review:ab AND review:it) NOT trial:ti,tt) OR ('we searched':ab AND (review:ti,tt OR review:it)) OR 'update review':ab OR (databases NEAR/5 searched):ab OR ((rat OR rats OR mouse OR mice OR swine OR porcine OR murine OR sheep OR lambs OR pigs OR piglets OR rabbit OR rabbits OR cat OR cats OR dog OR dogs OR cattle OR bovine OR monkey OR monkeys OR trout OR marmoset*):ti,tt AND 'animal experiment'/de) OR ('animal experiment'/de NOT ('human experiment'/de OR 'human'/de))  #18: #16 NOT #17  #19: #1 AND #14 AND #15 AND #18 | 2025.12.14  Items found:  568 |
| **Cochrane Library** | #1: [mh "Renal Dialysis"] OR [mh "Hemodiafiltration"] OR [mh "Renal Replacement Therapy"] OR (hemodialy* OR haemodialy* OR Hemodiafiltration OR hemofiltration OR haemofiltration OR dialysis OR dialyses OR "blood purification" OR "maintenance hemodialysis"):ti,ab,kw  #2: [mh "Complementary Therapies"] OR (nonpharmacologic* OR (non NEXT pharmacologic*) OR (non NEXT drug*) OR nondrug*):ti,ab,kw  #3: [mh "Acupressure"] OR (Acupressure OR acupoint* OR "auricular point" OR "ear point" OR shiatsu):ti,ab,kw  #4: [mh "Acupuncture Therapy"] OR (acupuncture OR electroacupuncture OR (needle NEXT therap*)):ti,ab,kw  #5: [mh "Aromatherapy"] OR (aromatherap* OR (aroma NEXT therap*) OR lavender OR (essential NEXT oil*) OR "lavender oil"):ti,ab,kw  #6: [mh "Massage"] OR (Massage OR tuina OR (manual NEXT therap*) OR rubbing OR effleurage):ti,ab,kw  #7: (reflexology OR "foot massage" OR "foot zone" OR "zone therapy" OR reflexotherap*):ti,ab,kw  #8: [mh "Exercise"] OR [mh "Exercise Therapy"] OR [mh "Resistance Training"] OR [mh "Yoga"] OR [mh "Tai Ji"] OR [mh "Walking"] OR [mh "Qigong"] OR (exercis* OR "aerobic training" OR "Resistance Training" OR "strength training" OR "weight training" OR "physical training" OR (physical NEXT activit*) OR "intradialytic training" OR walk* OR cycl* OR bicycle OR treadmill OR ergometer OR Yoga OR pilates OR "tai chi" OR Qigong OR baduanjin):ti,ab,kw  #9: [mh "Mind-Body Therapies"] OR [mh "Mindfulness"] OR [mh "Meditation"] OR [mh "Relaxation Therapy"] OR ("mind-body" OR mindful* OR meditati* OR relaxation):ti,ab,kw  #10: [mh "Psychotherapy"] OR [mh "Behavior Therapy"] OR [mh "Cognitive Behavioral Therapy"] OR ((psycholog* AND (therap* OR intervention* OR treatment* OR counsel*)) OR (behavioral NEXT intervention*) OR (behavioural NEXT intervention*) OR (cognitive NEXT behavioral NEXT therap*) OR (cognitive NEXT behavioural NEXT therap*) OR (cognitive NEXT behavioral NEXT treatment*) OR (cognitive NEXT behavioural NEXT treatment*) OR CBT):ti,ab,kw  #11: [mh "Music Therapy"] OR (Music*):ti,ab,kw  #12: [mh "Health Education"] OR [mh "Patient Education as Topic"] OR ((education NEXT program*) OR "patient education" OR "Health Education" OR (education NEXT intervention*) OR (educational NEXT intervention*) OR "self management" OR "self care"):ti,ab,kw  #13: [mh "Telemedicine"] OR [mh "Remote Consultation"] OR [mh "Telerehabilitation"] OR [mh "Internet"] OR [mh "Internet-Based Intervention"] OR [mh "Therapy, computer assisted"] OR [mh "Mobile Applications"] OR [mh "Smartphone"] OR [mh "Cell Phone"] OR [mh "Text Messaging"] OR [mh "Wearable Electronic Devices"] OR [mh "Fitness Trackers"] OR [mh "Wireless Technology"] OR [mh "Virtual Reality"] OR [mh "Exergaming"] OR [mh "Video Games"] OR [mh "Robotics"] OR [mh "Artificial Intelligence"] OR (telemedicine OR telehealth* OR telecare OR ehealth OR mhealth OR "digital health" OR (remote NEXT consult*) OR (video NEXT consult*) OR teleconsult* OR (virtual NEXT visit*) OR (video NEXT visit*) OR (mobile AND (app OR apps OR application*)) OR smartphone* OR "internet-based" OR "web-based" OR (online NEXT intervention*) OR (online NEXT program*) OR wearable* OR pedometer* OR accelerometer* OR "virtual reality" OR exergame* OR (video NEXT game*) OR gamification OR robot* OR "artificial intelligence"):ti,ab,kw  #14: #2 OR #3 OR #4 OR #5 OR #6 OR #7 OR #8 OR #9 OR #10 OR #11 OR #12 OR #13  #15: [mh "Fatigue"] OR (fatigue* OR tiredness OR weary OR weariness OR exhaustion OR exhausted OR tired OR vitality OR lassitude):ti,ab,kw  #16: #1 AND #14 AND #15 | 2025.12.14  Items found:  493 |
| **CINAHL** | S1: (MH "Hemodialysis+") OR (MH "Hemodiafiltration") OR (MH "Renal Replacement Therapy+") OR TI ( hemodialy* OR haemodialy* OR Hemodiafiltration OR hemofiltration OR haemofiltration OR dialysis OR dialyses OR "blood purification" OR "maintenance hemodialysis" ) OR AB ( hemodialy* OR haemodialy* OR Hemodiafiltration OR hemofiltration OR haemofiltration OR dialysis OR dialyses OR "blood purification" OR "maintenance hemodialysis" )  S2: (MH "Alternative Therapies+") OR TI ( nonpharmacologic* OR "non pharmacologic*" OR "non drug*" OR nondrug* ) OR AB ( nonpharmacologic* OR "non pharmacologic*" OR "non drug*" OR nondrug* )  S3: (MH "Acupressure") OR TI ( acupressure OR acupoint* OR "auricular point" OR "ear point" OR shiatsu ) OR AB ( acupressure OR acupoint* OR "auricular point" OR "ear point" OR shiatsu )  S4: (MH "Acupuncture+") OR TI ( acupuncture OR electroacupuncture OR "needle therap*" ) OR AB ( acupuncture OR electroacupuncture OR "needle therap*" )  S5: (MH "Aromatherapy+") OR TI ( aromatherap* OR "aroma therap*" OR lavender OR "essential oil*" OR "lavender oil" ) OR AB ( aromatherap* OR "aroma therap*" OR lavender OR "essential oil*" OR "lavender oil" )  S6: (MH "Massage+") OR TI ( massage OR tuina OR "manual therap*" OR rubbing OR effleurage ) OR AB ( massage OR tuina OR "manual therap*" OR rubbing OR effleurage )  S7: (MH "Reflexology") OR TI ( reflexology OR "foot massage" OR "foot zone" OR "zone therapy" OR reflexotherap* ) OR AB ( reflexology OR "foot massage" OR "foot zone" OR "zone therapy" OR reflexotherap* )  S8: (MH "Exercise+") OR (MH "Therapeutic Exercise+") OR (MH "Resistance Training+") OR (MH "Yoga") OR (MH "Tai Chi") OR (MH "Walking+") OR (MH "Qigong") OR TI ( exercis* OR "aerobic training" OR "Resistance Training" OR "strength training" OR "weight training" OR "physical training" OR "physical activit*" OR "intradialytic training" OR walk* OR cycl* OR bicycle OR treadmill OR ergometer OR Yoga OR pilates OR "tai chi" OR Qigong OR baduanjin ) OR AB ( exercis* OR "aerobic training" OR "Resistance Training" OR "strength training" OR "weight training" OR "physical training" OR "physical activit*" OR "intradialytic training" OR "intradialytic exercise" OR walk* OR cycl* OR bicycle OR treadmill OR ergometer OR Yoga OR pilates OR "tai chi" OR Qigong OR baduanjin )  S9: (MH "Mind Body Techniques+") OR (MH "Mindfulness") OR (MH "Meditation") OR (MH "Relaxation Techniques") OR TI ( "mind-body" OR mindful* OR meditati* OR relaxation ) OR AB ( "mind-body" OR mindful* OR meditati* OR relaxation )  S10: (MH "Psychotherapy+") OR (MH "Behavior Therapy+") OR (MH "Cognitive Behavioral Therapy") OR TI ( ((psycholog* AND (therap* OR intervention* OR treatment* OR counsel*)) OR "behavioral intervention*" OR "behavioural intervention*" OR "cognitive behavioral therap*" OR "cognitive behavioural therap*" OR "cognitive behavioral treatment*" OR "cognitive behavioural treatment*" OR CBT) ) OR AB ( ((psycholog* AND (therap* OR intervention* OR treatment* OR counsel*)) OR "behavioral intervention*" OR "behavioural intervention*" OR "cognitive behavioral therap*" OR "cognitive behavioural therap*" OR "cognitive behavioral treatment*" OR "cognitive behavioural treatment*" OR CBT) )  S11: (MH "Music Therapy+") OR TI ( Music* ) OR AB ( Music* )  S12: (MH "Health Education+") OR (MH "Patient Education+") OR TI ( "education program*" OR "patient education" OR "health education" OR "education intervention*" OR "educational intervention*" OR "self management" OR "self care" ) OR AB ( "education program*" OR "patient education" OR "health education" OR "education intervention*" OR "educational intervention*" OR "self management" OR "self care" )  S13: (MH "Telehealth+") OR (MH "Remote Consultation") OR (MH "Telerehabilitation") OR (MH "Internet+") OR (MH "Web-based Interventions") OR (MH "Computer Assisted Therapy+") OR (MH "Mobile Applications") OR (MH "Smartphone") OR (MH "Text Messaging") OR (MH "Wearable Technology+") OR (MH "Virtual Reality") OR (MH "Video Games") OR (MH "Robotics") OR (MH "Artificial Intelligence+") OR TI ( telemedicine OR telehealth* OR telecare OR ehealth OR mhealth OR "digital health" OR "remote consult*" OR "video consult*" OR teleconsult* OR "virtual visit*" OR "video visit*" OR (mobile AND (app OR apps OR application*)) OR smartphone* OR "internet-based" OR "web-based" OR "online intervention*" OR "online program*" OR wearable* OR pedometer* OR accelerometer* OR "virtual reality" OR exergame* OR "video game*" OR gamification OR robot* OR "artificial intelligence" ) OR AB ( telemedicine OR telehealth* OR telecare OR ehealth OR mhealth OR "digital health" OR "remote consult*" OR "video consult*" OR teleconsult* OR "virtual visit*" OR "video visit*" OR (mobile AND (app OR apps OR application*)) OR smartphone* OR "internet-based" OR "web-based" OR "online intervention*" OR "online program*" OR wearable* OR pedometer* OR accelerometer* OR "virtual reality" OR exergame* OR "video game*" OR gamification OR robot* OR "artificial intelligence" )  S14: S2 OR S3 OR S4 OR S5 OR S6 OR S7 OR S8 OR S9 OR S10 OR S11 OR S12 OR S13  S15: (MH "Fatigue+") OR TI ( fatigue* OR tiredness OR weary OR weariness OR exhaustion OR exhausted OR tired OR vitality OR lassitude ) OR AB ( fatigue* OR tiredness OR weary OR weariness OR exhaustion OR exhausted OR tired OR vitality OR lassitude )  S16: ((MH randomized controlled trials+) OR (MH double‐blind studies) OR (MH single‐blind studies) OR (MH random assignment) OR (MH pretest‐posttest design) OR (MH cluster sample) OR (TI (randomised OR randomized)) OR (AB (random*)) OR (TI (trial)) OR (MH (sample size) AND AB (assigned OR allocated OR control)) OR (MH (placebos)) OR (PT (randomized controlled trial)) OR (AB (control W5 group)) OR (MH (crossover design) OR MH (comparative studies)) OR (AB (cluster W3 RCT))) NOT (((MH animals+) OR (MH (animal studies)) OR (TI (animal model*))) NOT (MH (human)))  S17: S1 AND S14 AND S15 AND S16 | 2025.12.14  Items found:  140 |
| **Web of Science** | #1: TS=("Renal Dialysis" OR "Renal Replacement Therapy" OR hemodialy* OR haemodialy* OR Hemodiafiltration OR hemofiltration OR haemofiltration OR dialysis OR dialyses OR "blood purification" OR "maintenance hemodialysis")  #2: TS=("Complementary Therapies" OR nonpharmacologic* OR "non pharmacologic*" OR "non drug*" OR nondrug*)  #3: TS=("Acupressure" OR acupoint* OR "auricular point" OR "ear point" OR shiatsu OR acupuncture OR electroacupuncture OR "needle therap*" OR aromatherap* OR "aroma therap*" OR lavender OR "essential oil*" OR "lavender oil" OR "Massage" OR tuina OR "manual therap*" OR rubbing OR effleurage OR reflexology OR "foot massage" OR "foot zone" OR "zone therapy" OR reflexotherap*)  #4: TS=("Exercise Therapy" OR "Resistance Training" OR "Yoga" OR "Tai Ji" OR "Qigong" OR exercis* OR "aerobic training" OR "strength training" OR "weight training" OR "physical training" OR "physical activit*" OR "intradialytic training" OR walk* OR cycl* OR bicycle OR treadmill OR ergometer OR pilates OR "tai chi" OR baduanjin)  #5: TS=("mind-body" OR mindful* OR meditati* OR relaxation OR "Psychotherapy" OR "Behavior Therapy" OR (psycholog* AND (therap* OR intervention* OR treatment* OR counsel*)) OR "behavioral intervention*" OR "behavioural intervention*" OR "cognitive behavioral therap*" OR "cognitive behavioural therap*" OR "cognitive behavioral treatment*" OR "cognitive behavioural treatment*" OR CBT)  #6: TS=("Music Therapy" OR Music* OR "Health Education" OR "Patient Education as Topic" OR "education program*" OR "patient education" OR "education intervention*" OR "educational intervention*" OR "self management" OR "self care")  #7: TS=("Telemedicine" OR "Remote Consultation" OR "Telerehabilitation" OR "Internet" OR "Therapy, computer assisted" OR "Cell Phone" OR "Text Messaging" OR "Fitness Trackers" OR "Wireless Technology" OR "Virtual Reality" OR "Artificial Intelligence" OR telehealth* OR telecare OR ehealth OR mhealth OR "digital health" OR "remote consult*" OR "video consult*" OR teleconsult* OR "virtual visit*" OR "video visit*" OR (mobile AND (app OR apps OR application*)) OR smartphone* OR "internet-based" OR "web-based" OR "online intervention*" OR "online program*" OR wearable* OR pedometer* OR accelerometer* OR exergame* OR "video game*" OR gamification OR robot*)  #8: #2 OR #3 OR #4 OR #5 OR #6 OR #7  #9: TS=(fatigue* OR tiredness OR weary OR weariness OR exhaustion OR exhausted OR tired OR vitality OR lassitude)  #10: TS=(random* OR "clinical trial*" OR "controlled trial*" OR placebo* OR "double-blind*" OR "single-blind*" OR "randomi?ed controlled trial*" OR "rct" OR "cluster random*" OR "controlled clinical trial*")  #11: #1 AND #8 AND #9 AND #10 | 2025.12.14  Items found:  250 |
