# Supplementary material for: Comparative efficacy of non-pharmacological interventions on fatigue in people undergoing hemodialysis: a systematic review and network meta-analysis
Source: Front Med (Lausanne). 2026 Aug 19;13:1862895. doi: 10.3389/fmed.2026.1862895 (PMC13533668; doi:10.3389/fmed.2026.1862895)
Supplement: Supplementary file 2 [file Table_2.docx]

Supplementary Table 2. Study-level classification of multicomponent interventions

| **Study** | **Components of the intervention** | **Final node assignment** | **Basis for classification** |
| --- | --- | --- | --- |
| Eroğlu 2022 | Benson relaxation technique delivered concurrently with music therapy. | Mind-body therapy | The intervention was organized around breathing, focused attention, and elicitation of the relaxation response. Music accompanied and facilitated the structured relaxation protocol; the principal therapeutic target was therefore psychophysiological regulation. |
| Mohammadpourhodki 2021 | Foot massage with lavender essential oil; foot massage with Citrus aurantium essential oil; foot massage alone. | Aromatherapy for the two essential-oil arms; manual therapy for the massage-only arm. | The study was explicitly designed to evaluate aromatherapy massage, and the massage-only group provided an active comparator with a similar massage procedure. Essential-oil exposure was therefore considered the principal therapeutic component distinguishing the two intervention arms, whereas the massage-only comparator was classified as manual therapy. |
| Varaei 2020 | Inhalation aromatherapy; aromatherapy massage with lavender and sweet-orange essential oils. | Aromatherapy | Essential-oil exposure was the defining component shared by both active arms. Massage was an additional delivery component in only one arm; both active arms were therefore assigned to and combined within the aromatherapy node. |
| Habibzadeh 2020 | Foot massage with chamomile oil, almond oil, or no oil; no-intervention control. | Manual therapy for the three massage arms; usual care for the no-intervention control. | The study was framed around foot massage, and the same massage procedure was delivered across all three active arms, including the no-oil arm. Oil type was treated as a variation or adjunct to the massage protocol rather than as a separate aromatherapy intervention; the three active arms were therefore classified as manual therapy and combined after classification. |
| Lazarus 2021 | Olive-oil massage using effleurage and kneading techniques. | Manual therapy | The protocol was primarily delivered through structured manual manipulation. Olive oil was treated as an adjunct or massage medium rather than as a separately estimable intervention component. |
| Chen 2011 | Cognitive behavioral therapy plus sleep-hygiene education versus sleep-hygiene education alone. | Cognitive behavioral therapy for the combination arm; brief behavioral intervention for the comparator | Sleep-hygiene education was provided to both groups. Structured cognitive behavioral therapy was the incremental component distinguishing the experimental arm, whereas sleep-hygiene education alone met the definition of a brief behavioral intervention. |
| Cho 2004 | Acupressure at predefined acupoints followed by brief lower-limb massage. | Acupoint stimulation | The intervention was centered on stimulation of prespecified acupoints for 12 minutes; the subsequent 3-minute massage was an adjunctive component. Acupoint stimulation was therefore considered predominant. |

Notes: Multicomponent interventions were defined as treatment packages combining distinguishable non-pharmacological components that could potentially correspond to different network nodes, or as add-on designs involving an active background intervention. Combinations of techniques falling entirely within the same node, such as aerobic plus resistance exercise, were not considered multicomponent for this classification. Original study arms assigned to the same node were combined only after node classification.

Two reviewers independently classified multicomponent interventions using a predominant-component rule. Node assignment was based primarily on the intervention’s stated therapeutic target and the operational definition of each node. Comparator structure, shared components, distinguishing components, and the relative duration and intensity of individual components were used as supporting criteria. An added component was assigned to a separate node only when it represented the intended principal therapeutic component rather than a delivery medium or protocol variation. Disagreements were resolved through consensus or consultation with a third reviewer.
