# Supplementary material for: Comparative efficacy of non-pharmacological interventions on fatigue in people undergoing hemodialysis: a systematic review and network meta-analysis
Source: Front Med (Lausanne). 2026 Aug 19;13:1862895. doi: 10.3389/fmed.2026.1862895 (PMC13533668; doi:10.3389/fmed.2026.1862895)
Supplement: Supplementary file 3 [file Table_3.docx]

Supplementary Table 3. Distribution of fatigue-scale categories and exploratory sensitivity analysis restricted to FSS- and PFS-based trials

Panel A. Distribution of fatigue-scale categories across network nodes

| **Network node** | **FSS** | **PFS** | **MFI** | **Other** | **Total node-trial occurrences** |
| --- | --- | --- | --- | --- | --- |
| Usual care | 8 | 9 | 2 | 10 | 29 |
| Sham control | 1 | 4 | 1 | 2 | 8 |
| Exercise | 1 | 1 | 2 | 5 | 9 |
| Mind-body therapy | 1 | 4 | 1 | 1 | 7 |
| Cognitive behavioral therapy | 1 | 0 | 0 | 1 | 2 |
| Brief behavioral intervention | 1 | 0 | 1 | 2 | 4 |
| Aromatherapy | 4 | 2 | 0 | 2 | 8 |
| Manual therapy | 4 | 1 | 0 | 2 | 7 |
| Acupoint stimulation | 0 | 4 | 1 | 1 | 6 |
| Non-invasive brain stimulation | 1 | 0 | 0 | 0 | 1 |

Panel B. Distribution of fatigue-scale categories by geographic region and publication period

| **Fatigue-scale category** | **Total trials** | **Iran/Türkiye** | **Other regions** | **≤2021** | **>2021** |
| --- | --- | --- | --- | --- | --- |
| FSS | 11 | 8 | 3 | 7 | 4 |
| PFS | 11 | 9 | 2 | 7 | 4 |
| MFI | 4 | 4 | 0 | 1 | 3 |
| Other | 13 | 6 | 7 | 7 | 6 |
| Total | 39 | 27 | 12 | 22 | 17 |

Panel C. Feasibility and results of the FSS/PFS-restricted sensitivity analysis

| **Restricted dataset** | **Trials** | **Participants** | **Analytic arms** | **Nodes** | **Network structure / analytical decision** |
| --- | --- | --- | --- | --- | --- |
| All FSS/PFS-based trials | 22 | 1848 | 47 | 10 | Two disconnected components; a unified 10-node NMA was not statistically valid and was not forced. |
| Main connected component | 21 | 1776 | 45 | 8 | One connected component after excluding the isolated Chen 2011 CBT–brief behavioral intervention comparison; exploratory random-effects NMA conducted. |

Exploratory estimates from the eight-node main connected component

| **Intervention versus usual care** | **SMD (95% CI)** | **P value** |
| --- | --- | --- |
| Sham control | −0.30 (−1.05 to 0.45) | 0.433 |
| Exercise | −1.09 (−2.02 to −0.17) | 0.021 |
| Mind-body therapy | −1.83 (−2.45 to −1.21) | <0.001 |
| Aromatherapy | −1.24 (−1.83 to −0.65) | <0.001 |
| Manual therapy | −0.73 (−1.34 to −0.12) | 0.019 |
| Acupoint stimulation | −0.91 (−1.65 to −0.17) | 0.016 |
| Non-invasive brain stimulation | −1.58 (−3.24 to 0.07) | 0.061 |

**Notes:** Panel A reports the number of trials involving each node within each fatigue-scale category; a multi-arm trial can contribute to more than one node. Trial counts in Panel B are study-level counts. The FSS/PFS-restricted evidence network separated into two disconnected components. Cognitive behavioral therapy and brief behavioral intervention formed an isolated component through Chen 2011 and therefore could not be compared with usual care within the restricted network. A unified 10-node model was not forced. The estimates shown are from the exploratory eight-node main connected component and should not be interpreted as a replacement for the primary analysis. Negative SMDs favor the intervention.

**Abbreviations:** CBT, cognitive behavioral therapy; CI, confidence interval; FSS, Fatigue Severity Scale; MFI, Multidimensional Fatigue Inventory; NMA, network meta-analysis; PFS, Piper Fatigue Scale; SMD, standardized mean difference.
