# Supplementary material for: Comparative efficacy of non-pharmacological interventions on fatigue in people undergoing hemodialysis: a systematic review and network meta-analysis
Source: Front Med (Lausanne). 2026 Aug 19;13:1862895. doi: 10.3389/fmed.2026.1862895 (PMC13533668; doi:10.3389/fmed.2026.1862895)
Supplement: Supplementary file 4 [file Table_4.docx]

Supplementary Table 4. Characteristics of included studies, analytic comparisons, and final node assignments

| **ID** | **Study** | **Country** | **Final analytic comparison / retained arms** | **Analytic N** | **Mean age, years** | **Dialysis vintage, months** | **Intervention details** | **Fatigue scale** | **Overall RoB 2** |
| --- | --- | --- | --- | --- | --- | --- | --- | --- | --- |
| 1 | Sung 2025 | China | Acupoint stimulation (n=35) vs. Sham control (n=36) | 71 | 59.28 | 85.69 | Laser acupuncture; 6 weeks; 3 sessions/week; 5 min/session. | Fatigue Visual Analog Scale | Low |
| 2 | Sharma 2025 | Nepal | Brief behavioral intervention (n=63) vs. Usual care (n=63) | 126 | 43.42 | NR | Energy-conservation education; 12 weeks; four sessions (three individual sessions plus one booster); 30-45 min/session. | Fatigue Symptom Inventory | High |
| 3 | Nakoui 2025 | Iran | Exercise (n=34) vs. Usual care (n=17) | 51 | NR | NR | Aerobic and resistance training arms combined within the exercise node; 8 weeks; 3 sessions/week. | Fatigue Severity Scale | Some concerns |
| 4 | Mendes 2025 | Brazil | Non-invasive brain stimulation (n=17) vs. Sham control (n=17) | 34 | 53.7 | NR | Transcranial direct current stimulation; 5 weeks; 3 sessions/week; 20 min/session. | Fatigue Severity Scale | Low |
| 5 | Hasanzadeh 2025 | Iran | Mind-body therapy (n=34) vs. Exercise (n=32) | 66 | 48.12 | 51.82 | Progressive muscle relaxation: 4 weeks, twice daily, 50-60 min/session; physical exercise: 4 weeks, 4 sessions/week. | Multidimensional Fatigue Inventory | Some concerns |
| 6 | Feng 2025 | China | Exercise (n=28) vs. Usual care (n=30) | 58 | 56.78 | 60.48 | Intradialytic elastic-band exercise; 12 weeks; 3 sessions/week; at least 30 min/session. | Fatigue Scale-14 | Some concerns |
| 7 | Başer Akın 2025 | Turkiye | Mind-body therapy (n=37) vs. Usual care (n=37) | 74 | NR | NR | Reiki; 5 weeks; 2 sessions/week (10 sessions); approximately 40 min/session. | Piper Fatigue Scale | Some concerns |
| 8 | Asgari 2025 | Iran | Acupoint stimulation (n=27) vs. Sham control (n=28) | 55 | 50.8 | 47.08 | Self-auriculotherapy; 6 weeks; 3 supervised sessions/week, with self-pressure four times daily for 1-2 min per point. | Multidimensional Fatigue Inventory | Some concerns |
| 9 | Sahebkar 2024 | Iran | Brief behavioral intervention (n=34) vs. Usual care (n=32) | 66 | 56.51 | 39.97 | Humor therapy; 3 weeks; 2 sessions/week; 60 min/session. | Fatigue Symptom Inventory | Some concerns |
| 10 | Alishahi 2024 | Iran | Brief behavioral intervention (n=36) vs. Usual care (n=36) | 72 | 44.05 | 53.85 | Recreational therapy; 8 weeks; 30 min daily. | Multidimensional Fatigue Inventory | Some concerns |
| 11 | Bayülgen 2023 | Turkiye | Mind-body therapy (n=30) vs. Usual care (n=31) | 61 | 53.53 | 63.11 | Reiki; 4 weeks; 3 sessions/week; 36-40 min/session. | Fatigue Severity Scale | Some concerns |
| 12 | Rahimi 2022 | Iran | Aromatherapy (n=31) vs. Sham control (n=31) | 62 | 58.78 | 71.4 | Lavender aromatherapy by inhalation; 4 weeks; 3 sessions/week; 2 min/session. | Piper Fatigue Scale | High |
| 13 | Malini 2022 | Indonesia | Exercise (n=23) vs. Usual care (n=24) | 47 | 52.28 | 22.24 | Intradialytic range-of-motion exercise; 8 weeks; 2 sessions/week; 30 min/session. | Fatigue Assessment Scale | Some concerns |
| 14 | Kumar 2022 | Pakistan | Exercise (n=13) vs. Usual care (n=13) | 26 | 44.87 | 54.54 | Home-based walking plus resistance exercise; 6 weeks; 3 sessions/week; 20-30 min/session. | Fatigue Assessment Scale | Some concerns |
| 15 | Eroğlu 2022 | Turkiye | Mind-body therapy (n=30) vs. Usual care (n=31) | 61 | 55.39 | NR | Benson relaxation combined with music; 8 weeks; 2 sessions/week; 20 min/session. | Piper Fatigue Scale | Some concerns |
| 16 | Elahi 2022 | Iran | Aromatherapy (n=31) vs. Usual care (n=37) | 68 | 49.74 | NR | Rosa damascena oil aromatherapy by inhalation; 4 weeks; nightly during sleep. | Fatigue Severity Scale | Some concerns |
| 17 | Biçer 2022 | Turkiye | Acupoint stimulation (n=67) vs. Sham control (n=68) | 135 | 64.9 | 71.03 | Body acupressure; 4 weeks; 3 sessions/week; 60 min/session. | Piper Fatigue Scale | High |
| 18 | Picariello 2021 | United Kingdom | Cognitive behavioral therapy (n=11) vs. Usual care (n=7) | 18 | 56.41 | 48.92 | Cognitive behavioral therapy for fatigue; 4-6 weeks; 3-5 sessions (face-to-face and telephone delivery). | Chalder Fatigue Questionnaire | High |
| 19 | Mohammadpourhodki 2021 | Iran | Aromatherapy (n=70) vs. Manual therapy (n=35) | 105 | 52.96 | 41.33 | Lavender and Citrus aurantium aromatherapy-massage arms combined within the aromatherapy node versus massage alone; 4 weeks; 3 sessions/week; 20 min/session. | Fatigue Severity Scale | Some concerns |
| 20 | Huang 2021 | China | Exercise (n=40) vs. Usual care (n=43) | 83 | 57.45 | NR | Breathing-based leg exercise during hemodialysis; 12 weeks; 3 sessions/week; 15 min/session. | Hemodialysis-Related Fatigue Scale | Some concerns |
| 21 | Çeçen 2021 | Turkiye | Manual therapy (n=54) vs. Usual care (n=28) | 82 | 56.12 | 65.09 | Hand and foot massage arms combined within the manual-therapy node; 4 weeks; 3 sessions/week; 8 min/session. | Visual Analogue Scale for Fatigue | Some concerns |
| 22 | Varaei 2020 | Iran | Aromatherapy (n=64) vs. Usual care (n=32) | 96 | NR | NR | Inhalation aromatherapy and aromatherapy massage combined within the aromatherapy node; 8 weeks; 3 sessions/week; 10-20 min/session. | Rhoten Fatigue Scale | Some concerns |
| 23 | Salehi 2020 | Iran | Exercise (n=20) vs. Usual care (n=17) | 37 | 56.35 | 40.49 | Exercise during hemodialysis; 12 weeks; 2 sessions/week; 20 min/session. | Multidimensional Fatigue Inventory | High |
| 24 | Lazarus 2021 | India | Manual therapy (n=100) vs. Usual care (n=100) | 200 | NR | NR | Olive-oil massage; 8 weeks; twice weekly during hemodialysis. | Fatigue Severity Scale | High |
| 25 | Kaplan Serin 2020 | Turkiye | Mind-body therapy (n=48) vs. Usual care (n=48) | 96 | 44.45 | NR | Progressive relaxation exercise; 6 weeks; at least twice weekly with the researcher plus daily home practice; 30-40 min/session. | Piper Fatigue Scale | Some concerns |
| 26 | Habibzadeh 2020 | Iran | Manual therapy (n=90) vs. Usual care (n=30) | 120 | 55.2 | NR | Foot massage arms combined within the manual-therapy node; 8 weeks; 3 sessions/week; 20 min/session. | Fatigue Severity Scale | Some concerns |
| 27 | Karadag 2019 | Turkiye | Aromatherapy (n=30) vs. Usual care (n=30) | 60 | 51.1 | 48.83 | Aromatherapy; 30 days; 2-3 sessions/week; 20 min/session. | Fatigue Severity Scale | High |
| 28 | Sharifi 2018 | Iran | Manual therapy (n=45) vs. Usual care (n=43) | 88 | 48.98 | 41.33 | Foot reflexology; three consecutive dialysis sessions over 1 week; 30 min/session. | Fatigue Severity Scale | High |
| 29 | Muz 2017 | Turkiye | Aromatherapy (n=27) vs. Usual care (n=35) | 62 | 56.21 | 6.26 | Aromatherapy by inhalation; 4 weeks; nightly before sleep; 2 min/session. | Piper Fatigue Scale | High |
| 30 | Biçer 2017 | Turkiye | Aromatherapy (n=25) vs. Sham control (n=25) | 50 | NR | NR | Aromatherapy by inhalation versus placebo aromatherapy; 1 week; 3 sessions/week; 5 min/session. | Brief Fatigue Inventory | High |
| 31 | Ünal 2016 | Turkiye | Manual therapy (n=70) vs. Usual care (n=35) | 105 | 54.33 | NR | Foot reflexology and back massage arms combined within the manual-therapy node; 4 weeks; 2 sessions/week; 30 min/session. | Visual Analogue Scale for Fatigue | Some concerns |
| 32 | Bagheri-Nesami 2016 | Iran | Aromatherapy (n=29) vs. Usual care (n=30) | 59 | 60.79 | 42.16 | Lavender aromatherapy; 4 weeks; 3 sessions/week; 10 min/session. | Fatigue Severity Scale | Some concerns |
| 33 | Amini 2016 | Iran | Mind-body therapy (n=33) vs. Exercise (n=32) vs. Usual care (n=35) | 100 | 55.23 | NR | Progressive muscle relaxation and aerobic exercise; 8 weeks; daily. | Piper Fatigue Scale | Some concerns |
| 34 | Sabouhi 2013 | Iran | Acupoint stimulation (n=32) vs. Usual care (n=32) vs. Sham control (n=32) | 96 | 54.37 | NR | Acupressure versus usual care and sham acupressure; 4 weeks; 3 sessions/week; 20 min/session. | Piper Fatigue Scale | Some concerns |
| 35 | Özdemir 2013 | Turkiye | Manual therapy (n=40) vs. Usual care (n=40) | 80 | 48.55 | NR | Foot reflexology; 1 week; 3 sessions; 30 min/session. | Piper Fatigue Scale | Some concerns |
| 36 | Chen 2011 | China | Cognitive behavioral therapy (n=37) vs. Brief behavioral intervention (n=35) | 72 | 58 | 70.8 | Cognitive behavioral therapy versus a brief behavioral/psychoeducational comparator; 6 weeks; 3 sessions/week; 30 min/session. | Fatigue Severity Scale | Some concerns |
| 37 | Yurtkuran 2007 | Turkiye | Mind-body therapy (n=19) vs. Exercise (n=18) | 37 | 39.50 | 20.85 | Modified yoga-based exercise versus exercise; 12 weeks; 2 sessions/week; 30 min/session. | Visual Analogue Scale | Some concerns |
| 38 | Tsay 2004 | China | Acupoint stimulation (n=35) vs. Usual care (n=36) vs. Sham control (n=35) | 106 | 58.16 | 50.06 | Acupressure versus usual care and sham control; 4 weeks; 3 sessions/week; 15 min/session. | Revised Piper Fatigue Scale | Some concerns |
| 39 | Cho 2004 | China | Acupoint stimulation (n=28) vs. Usual care (n=30) | 58 | 49.51 | 57.9 | Acupressure with massage; 4 weeks; 3 sessions/week; 15 min/session. | Revised Piper Fatigue Scale | Some concerns |

Notes: Analytic N is the number of participants included in the final fatigue analysis across retained arms. Original study groups assigned to the same network node were combined before analysis in accordance with the Cochrane Handbook. The primary analysis used fatigue outcomes assessed at completion of the planned intervention; later follow-up assessments reported by Bayülgen et al., Eroğlu et al., and Salehi et al. were not pooled with end-of-intervention outcomes. NR, not reported; CBT, cognitive behavioral therapy; RoB 2, revised Cochrane risk-of-bias tool. Study IDs 1–39 correspond to references 1–39 listed below, respectively.

1.	Sung RY, Yeh ML, Chen FP, Lo MY, Yu J. Laser acupuncture improves post-dialysis fatigue and sleep quality independent of time-varying dialysis and depression symptoms: a double-blind, randomized, placebo-controlled trial. *BMC Complementary Med Ther*. 2025;26(1):19. doi:10.1186/s12906-025-05203-3

2.	Sharma S, Alexander KE, Green T, Wu ML, Bonner A. Energy conservation education intervention for people with end-stage kidney disease receiving haemodialysis (EVEREST): a two-arm parallel group study. *Int J Nurs Stud*. 2025;166:105032. doi:10.1016/j.ijnurstu.2025.105032

3.	Nakoui N, Ilbeigi S, Ahmadi MM, Saber A. Comparison of the effect of aerobic and resistance training on fatigue, quality of life and biochemical factors in hemodialysis patients. *Sci Rep*. 2025;15(1):10052. doi:10.1038/s41598-025-94257-x

4.	Mendes EF, Silva-Filho E, Holanda JB, et al. Transcranial direct current stimulation over motor cortex improves pain in end stage renal disease a randomized controlled trial. *Sci Rep*. 2025;15(1):21943. doi:10.1038/s41598-025-96415-7

5.	Hasanzadeh M, Miri K, Pourghaznein T, Vaghee S, Azhari A, AfzalAghaee M. The effect of progressive muscle relaxation and physical exercises on subjective well-being and fatigue of patients receiving hemodialysis: a randomized clinical trial. *Sci Rep*. 2025;15(1):43440. doi:10.1038/s41598-025-27302-4

6.	Feng X, Sun J, Wang Z, et al. The impact of intradialytic elastic band exercise on physical and cognitive abilities in patients on maintenance hemodialysis: a randomized controlled trial. *Ren Fail*. 2025;47(1):2482124. doi:10.1080/0886022X.2025.2482124

7.	Başer Akın E, Mollaoğlu M. Investigation of the effect of reiki on pain, fatigue, and itching in hemodialysis patients: randomized controlled trial. *Hemodial Int, Int Symp Home Hemodial*. 2025;29(2):201-213. doi:10.1111/hdi.13203

8.	Asgari F, Aghajani M, Nia SM. The effect of self-auriculotherapy on the severity of restless leg syndrome, sleep quality, and fatigue in hemodialysis patients: a randomized controlled clinical trial. *Explore*. 2025;21(5):103205. doi:10.1016/j.explore.2025.103205

9.	Sahebkar M, Ansari M, Attarzadeh F, Borzoee F. Evaluating the effects of humor therapy on fatigue levels of hemodialysis patients: a single-blind, randomized clinical trial study. *J Res Med Sci*. 2024;29:56. doi:10.4103/jrms.jrms_17_23

10.	Alishahi M, Mazloum SR, Mohajer S, Namazinia M. The effect of recreational therapy application on fatigue in hemodialysis patients: a randomized clinical trial. *BMC Nephrol*. 2024;25(1):368. doi:10.1186/s12882-024-03807-4

11.	Yeşil Bayülgen M, Gün M. The effect of reiki on fatigue and comfort in hemodialysis patients. *Explore*. 2023;19(4):553-560. doi:10.1016/j.explore.2022.12.009

12.	Rahimi E, Sedighi Chafjiri A, Hasavari F, Kazem Nezhad Leyli E, Naseri M, Khosravi M. Evaluation of the effect of lavender aroma on fatigue among hemodialysis patients. *Holist Nurs Pract*. 2022;36(2):76-84. doi:10.1097/HNP.0000000000000501

13.	Malini H, Forwaty E, Cleary M, Visentin D, Oktarina E, Lenggogeni DP. The effect of intradialytic range of motion exercise on dialysis adequacy and fatigue in hemodialysis patients. *J Nurs Res*. 2022;30(4):e221. doi:10.1097/jnr.0000000000000506

14.	Kumar N, Sheraz S, Pereira FA, Razzaq A, Angela C, Saad SM. Effects of home-based exercise program on physical functioning of hemodialysis patients: a randomized controlled trial. *Healthc Low Resour Settings*. 2022;10(1):56. doi:10.4081/hls.2022.10499

15.	Eroglu H, Gok Metin Z. Benson relaxation technique combined with music therapy for fatigue, anxiety, and depression in hemodialysis patients: a randomized controlled trial. *Holist Nurs Pract*. 2022;36(3):139-148. doi:10.1097/HNP.0000000000000509

16.	Elahi B, Parvan K, Jabbarzadeh Tabrizi F, Sarbakhsh P, Gharekhani A, Roshangar F. Effect of rosa damascena oil aromatherapy on fatigue severity in patients receiving hemodialysis: a randomized controlled trial. *Crescent J Med Biol Sci*. 2022;9(3):147-152. doi:10.34172/cjmb.2022.25

17.	Biçer S, Taşci S. The effect of body acupressure on blood pressure and fatigue levels in individuals suffering from hypotension during hemodialysis: a randomized controlled trial. *Altern Ther Health Med*. 2022;28(2):6-16.

18.	Picariello F, Moss-Morris R, Norton S, et al. Feasibility trial of cognitive behavioral therapy for fatigue in hemodialysis (BReF intervention). *J Pain Symptom Manage*. 2021;61(6):1234-1246.e5. doi:10.1016/j.jpainsymman.2020.10.005

19.	Mohammadpourhodki R, Sadeghnezhad H, Ebrahimi H, Basirinezhad MH, Maleki M, Bossola M. The effect of aromatherapy massage with lavender and citrus aurantium essential oil on quality of life of patients on chronic hemodialysis: a parallel randomized clinical trial study. *J Pain Symptom Manage*. 2021;61(3):456-463.e1. doi:10.1016/j.jpainsymman.2020.08.032

20.	Huang HY, Hung KS, Yeh ML, Chou HL, Yeh AL, Liao TY. Breathing-based leg exercises during hemodialysis improve quality of life: a randomized controlled trial. *Clin Rehabil*. 2021;35(8):1175-1184. doi:10.1177/02692155211000738

21.	Çeçen S, Lafcı D. The effect of hand and foot massage on fatigue in hemodialysis patients: a randomized controlled trial. *Complement Ther Clin Pract*. 2021;43:101344. doi:10.1016/j.ctcp.2021.101344

22.	Varaei S, Jalalian Z, Yekani Nejad MS, Shamsizadeh M. Comparison the effects of inhalation and massage aromatherapy with lavender and sweet orange on fatigue in hemodialysis patients: a randomized clinical trial. *J Complement Integr Med*. 2020;18(1):193-200. doi:10.1515/jcim-2018-0137

23.	Salehi F, Dehghan M, Mangolian Shahrbabaki P, Ebadzadeh MR. Effectiveness of exercise on fatigue in hemodialysis patients: a randomized controlled trial. *BMC Sports Sci Med Rehabil*. 2020;12:19. doi:10.1186/s13102-020-00165-0

24.	Lazarus ER, Deva Amirtharaj A, Jacob D, Chandrababu R, Isac C. The effects of an olive-oil massage on hemodialysis patients suffering from fatigue at a hemodialysis unit in southern India - a randomized controlled trial. *J Complement Integr Med*. 2020;18(2):397-403. doi:10.1515/jcim-2019-0338

25.	Kaplan Serin E, Ovayolu N, Ovayolu Ö. The effect of progressive relaxation exercises on pain, fatigue, and quality of life in dialysis patients. *Holist Nurs Pract*. 2020;34(2):121-128. doi:10.1097/HNP.0000000000000347

26.	Habibzadeh H, Wosoi Dalavan O, Alilu L, Wardle J, Khalkhali H, Nozad A. Effects of foot massage on severity of fatigue and quality of life in hemodialysis patients: a randomized controlled trial. *Int J Community Based Nurs Midwifery*. 2020;8(2):92-102. doi:10.30476/IJCBNM.2020.81662.0

27.	Karadag E, Samancioglu Baglama S. The effect of aromatherapy on fatigue and anxiety in patients undergoing hemodialysis treatment: a randomized controlled study. *Holist Nurs Pract*. 2019;33(4):222-229. doi:10.1097/HNP.0000000000000334

28.	Sharifi S, Navidian A, Jahantigh M, Lori AS. *Investigating the Impact of Foot Reflexology on Severity of Fatigue in Patients Undergoing Hemodialysis: A Clinical Trial Study*. Medical - Surgical Nursing Journal; 2018:e81634. doi:10.5812/msnj.81634

29.	Muz G, Taşcı S. Effect of aromatherapy via inhalation on the sleep quality and fatigue level in people undergoing hemodialysis. *Appl Nurs Res: ANR*. 2017;37:28-35. doi:10.1016/j.apnr.2017.07.004

30.	Bicer S, Demir G. The effect of aromatherapy inhalation on fatigue level in individuals undergoing hemodialysis therapy. *Int J Caring Sci*. 2017;10(1):161.

31.	Unal KS, Balci Akpinar R. The effect of foot reflexology and back massage on hemodialysis patients’ fatigue and sleep quality. *Complement Ther Clin Pract*. 2016;24:139-144. doi:10.1016/j.ctcp.2016.06.004

32.	Bagheri-Nesami M, Shorofi SA, Nikkhah A, Espahbodi F, Ghaderi Koolaee FS. The effects of aromatherapy with lavender essential oil on fatigue levels in haemodialysis patients: a randomized clinical trial. *Complement Ther Clin Pract*. 2016;22:33-37. doi:10.1016/j.ctcp.2015.12.002

33.	Amini E, Goudarzi I, Masoudi R, Ahmadi A, Momeni A. Effect of progressive muscle relaxation and aerobic exercise on anxiety, sleep quality, and fatigue in patients with chronic renal failure undergoing hemodialysis. *Int J Pharm Clin Res*. 2016;8:1634-1639.

34.	Sabouhi F, Kalani L, Valiani M, Mortazavi M, Bemanian M. Effect of acupressure on fatigue in patients on hemodialysis. *Iran J Nurs Midwifery Res*. 2013;18(6):429-434.

35.	Ozdemir G, Ovayolu N, Ovayolu O. The effect of reflexology applied on haemodialysis patients with fatigue, pain and cramps. *Int J Nurs Pract*. 2013;19(3):265-273. doi:10.1111/ijn.12066

36.	Chen HY, Cheng IC, Pan YJ, et al. Cognitive-behavioral therapy for sleep disturbance decreases inflammatory cytokines and oxidative stress in hemodialysis patients. *Kidney Int*. 2011;80(4):415-422. doi:10.1038/ki.2011.151

37.	Yurtkuran M, Alp A, Yurtkuran M, Dilek K. A modified yoga-based exercise program in hemodialysis patients: a randomized controlled study. *Complement Ther Med*. 2007;15(3):164-171. doi:10.1016/j.ctim.2006.06.008

38.	Tsay SL. Acupressure and fatigue in patients with end-stage renal disease-a randomized controlled trial. *Int J Nurs Stud*. 2004;41(1):99-106. doi:10.1016/s0020-7489(03)00079-8

39.	Cho YC, Tsay SL. The effect of acupressure with massage on fatigue and depression in patients with end-stage renal disease. *J Nurs Res: JNR*. 2004;12(1):51-59. doi:10.1097/01.jnr.0000387488.63438.9a
