# Supplementary material for: Comparative efficacy of non-pharmacological interventions on fatigue in people undergoing hemodialysis: a systematic review and network meta-analysis
Source: Front Med (Lausanne). 2026 Aug 19;13:1862895. doi: 10.3389/fmed.2026.1862895 (PMC13533668; doi:10.3389/fmed.2026.1862895)
Supplement: Supplementary file 5 [file Table_5.docx]

Supplementary Table 5. Assessment of the transitivity assumption

| **Potential effect modifier** | **Availability** | **Observed distribution** | **Assessment across comparison designs** | **Judgment** |
| --- | --- | --- | --- | --- |
| Participant age | 34/39 trials | Mean age 39.50–64.90 years | Overlapping ranges across the more frequently studied nodes; assessment limited for sparse nodes | No major imbalance identified |
| Dialysis vintage | 23/39 trials | Mean 6.26–85.69 months | Unavailable in 16 trials, limiting comparison across designs | Residual concern |
| Intervention duration | 39/39 trials | ≤6 weeks: 26 trials; >6 weeks: 13 trials | Both categories represented across the network; meta-regression P=0.366 | No statistical effect modification detected |
| Geographic setting | 39/39 trials | Iran/Türkiye: 27 trials; other countries: 12 trials | Uneven distribution; meta-regression P=0.934 with limited power | Residual concern and limited generalizability |
| Fatigue scale type | 39/39 trials | FSS: 11; PFS: 11; MFI: 4; other: 13 | Uneven distribution; global meta-regression χ²=34.60, df=18, P=0.011 | Important concern |
| Overall risk of bias | 39/39 trials | Low: 2; some concerns: 27; high: 10 | High-risk trials occurred across several nodes; sensitivity analysis retained most effect directions | Residual concern |

Overall judgment: Transitivity was considered plausible but not fully assured because of fatigue-scale differences, geographic concentration, sparse treatment nodes, and incomplete reporting of dialysis vintage.
