# Supplementary material for: Comparative efficacy of non-pharmacological interventions on fatigue in people undergoing hemodialysis: a systematic review and network meta-analysis
Source: Front Med (Lausanne). 2026 Aug 19;13:1862895. doi: 10.3389/fmed.2026.1862895 (PMC13533668; doi:10.3389/fmed.2026.1862895)
Supplement: Supplementary file 6 [file Table_6.docx]

Supplementary Table 6. Summary of inconsistency assessments

| **Level** | **Method/comparison** | **Result** | **Interpretation** |
| --- | --- | --- | --- |
| Global | Design-by-treatment interaction model | χ²=6.38; df=8; P=0.605 | No statistically significant global inconsistency detected |
| Local | Node-splitting analyses | All P>0.05 | No statistically significant direct–indirect disagreement detected |
| Largest discrepancy | Sham control versus usual care | Wide, overlapping confidence intervals; P>0.05 | Substantial uncertainty remained despite the non-significant test |

Notes: Statistical non-significance does not prove consistency or transitivity; results were interpreted with network geometry, confidence intervals, and clinical heterogeneity.
