# Supplementary material for: Comparative efficacy of non-pharmacological interventions on fatigue in people undergoing hemodialysis: a systematic review and network meta-analysis
Source: Front Med (Lausanne). 2026 Aug 19;13:1862895. doi: 10.3389/fmed.2026.1862895 (PMC13533668; doi:10.3389/fmed.2026.1862895)
Supplement: Supplementary file 7 [file Table_7.docx]

Supplementary Table 7. Exploratory univariable network meta-regression

| **Moderator** | **Test/statistic** | **P value** | **Interpretation** |
| --- | --- | --- | --- |
| Intervention duration (>6 vs ≤6 weeks) | Global test | 0.366 | No statistically significant effect modification |
| Geographic region (Iran/Türkiye vs other) | Global test | 0.934 | No statistically significant effect modification; limited power |
| Mean participant age | Global test | 0.751 | No statistically significant effect modification |
| Mean dialysis vintage | Global test | 0.279 | No statistically significant effect modification; 16 trials missing |
| Publication year (continuous) | Global test | 0.703 | No statistically significant effect modification |
| Publication period (>2021 vs ≤2021) | Global test | 0.844 | No statistically significant effect modification |
| Fatigue scale type | χ²=34.60; df=18 | 0.011 | Exploratory evidence of effect modification; interpret cautiously |

Notes: All models were univariable and exploratory. Non-significant findings do not exclude clinically relevant effect modification when evidence is sparse or incompletely reported.
