# Supplementary material for: Comparative efficacy of non-pharmacological interventions on fatigue in people undergoing hemodialysis: a systematic review and network meta-analysis
Source: Front Med (Lausanne). 2026 Aug 19;13:1862895. doi: 10.3389/fmed.2026.1862895 (PMC13533668; doi:10.3389/fmed.2026.1862895)
Supplement: Supplementary file 8 [file Table_8.docx]

Supplementary Table 8. Complete CINeMA assessments for all 45 network comparisons

| **Comparison** | **Direct studies** | **Within-study bias** | **Reporting bias** | **Indirectness** | **Imprecision** | **Heterogeneity** | **Incoherence** | **Confidence** |
| --- | --- | --- | --- | --- | --- | --- | --- | --- |
| Acupoint stimulation vs Sham control | 5 | Some concerns | Some concerns | No concerns | No concerns | Major concerns | No concerns | Very low |
| Acupoint stimulation vs Usual care | 3 | Some concerns | Some concerns | No concerns | No concerns | Major concerns | No concerns | Low |
| Aromatherapy vs Manual therapy | 1 | Some concerns | Some concerns | No concerns | Some concerns | Some concerns | No concerns | Very low |
| Aromatherapy vs Sham control | 2 | Major concerns | Some concerns | No concerns | No concerns | No concerns | No concerns | Very low |
| Aromatherapy vs Usual care | 5 | Some concerns | Some concerns | No concerns | No concerns | No concerns | No concerns | Moderate |
| Brief behavioral intervention vs Cognitive behavioral therapy | 1 | Some concerns | Some concerns | No concerns | Major concerns | No concerns | No concerns | Very low |
| Brief behavioral intervention vs Usual care | 3 | Some concerns | Some concerns | No concerns | No concerns | No concerns | No concerns | Moderate |
| Cognitive behavioral therapy vs Usual care | 1 | Major concerns | Some concerns | No concerns | No concerns | Major concerns | No concerns | Very low |
| Exercise vs Mind-body therapy | 3 | Some concerns | Some concerns | No concerns | No concerns | Major concerns | No concerns | Very low |
| Exercise vs Usual care | 7 | Some concerns | Some concerns | No concerns | No concerns | Major concerns | No concerns | Low |
| Manual therapy vs Usual care | 6 | Some concerns | Some concerns | No concerns | No concerns | Major concerns | No concerns | Low |
| Mind-body therapy vs Usual care | 5 | Some concerns | Some concerns | No concerns | No concerns | No concerns | No concerns | Moderate |
| Non-invasive brain stimulation vs Sham control | 1 | No concerns | Some concerns | No concerns | Some concerns | Some concerns | No concerns | Low |
| Sham control vs Usual care | 2 | Some concerns | Some concerns | No concerns | Major concerns | No concerns | No concerns | Low |
| Acupoint stimulation vs Aromatherapy | 0 | Some concerns | Some concerns | No concerns | Major concerns | No concerns | No concerns | Very low |
| Acupoint stimulation vs Brief behavioral intervention | 0 | Some concerns | Some concerns | No concerns | Some concerns | Some concerns | No concerns | Very low |
| Acupoint stimulation vs Cognitive behavioral therapy | 0 | Some concerns | Some concerns | No concerns | Major concerns | No concerns | No concerns | Very low |
| Acupoint stimulation vs Exercise | 0 | Some concerns | Some concerns | No concerns | Major concerns | No concerns | No concerns | Very low |
| Acupoint stimulation vs Manual therapy | 0 | Some concerns | Some concerns | No concerns | Major concerns | No concerns | No concerns | Very low |
| Acupoint stimulation vs Mind-body therapy | 0 | Some concerns | Some concerns | No concerns | Some concerns | Some concerns | No concerns | Very low |
| Acupoint stimulation vs Non-invasive brain stimulation | 0 | Some concerns | Some concerns | No concerns | Major concerns | No concerns | No concerns | Very low |
| Aromatherapy vs Brief behavioral intervention | 0 | Some concerns | Some concerns | No concerns | Major concerns | No concerns | No concerns | Very low |
| Aromatherapy vs Cognitive behavioral therapy | 0 | Some concerns | Some concerns | No concerns | Major concerns | No concerns | No concerns | Very low |
| Aromatherapy vs Exercise | 0 | Some concerns | Some concerns | No concerns | Major concerns | No concerns | No concerns | Very low |
| Aromatherapy vs Mind-body therapy | 0 | Some concerns | Some concerns | No concerns | Major concerns | No concerns | No concerns | Very low |
| Aromatherapy vs Non-invasive brain stimulation | 0 | Some concerns | Some concerns | No concerns | Major concerns | No concerns | No concerns | Very low |
| Brief behavioral intervention vs Exercise | 0 | Some concerns | Some concerns | No concerns | Some concerns | Some concerns | No concerns | Very low |
| Brief behavioral intervention vs Manual therapy | 0 | Some concerns | Some concerns | No concerns | No concerns | Major concerns | No concerns | Very low |
| Brief behavioral intervention vs Mind-body therapy | 0 | Some concerns | Some concerns | No concerns | Major concerns | No concerns | No concerns | Very low |
| Brief behavioral intervention vs Non-invasive brain stimulation | 0 | Some concerns | Some concerns | No concerns | Major concerns | No concerns | No concerns | Very low |
| Brief behavioral intervention vs Sham control | 0 | Some concerns | Some concerns | No concerns | No concerns | No concerns | No concerns | Low |
| Cognitive behavioral therapy vs Exercise | 0 | Some concerns | Some concerns | No concerns | Major concerns | No concerns | No concerns | Very low |
| Cognitive behavioral therapy vs Manual therapy | 0 | Some concerns | Some concerns | No concerns | Major concerns | No concerns | No concerns | Very low |
| Cognitive behavioral therapy vs Mind-body therapy | 0 | Some concerns | Some concerns | No concerns | Major concerns | No concerns | No concerns | Very low |
| Cognitive behavioral therapy vs Non-invasive brain stimulation | 0 | Some concerns | Some concerns | No concerns | Major concerns | No concerns | No concerns | Very low |
| Cognitive behavioral therapy vs Sham control | 0 | Some concerns | Some concerns | No concerns | No concerns | Major concerns | No concerns | Very low |
| Exercise vs Manual therapy | 0 | Some concerns | Some concerns | No concerns | Major concerns | No concerns | No concerns | Very low |
| Exercise vs Non-invasive brain stimulation | 0 | Some concerns | Some concerns | No concerns | Major concerns | No concerns | No concerns | Very low |
| Exercise vs Sham control | 0 | Some concerns | Some concerns | No concerns | No concerns | Major concerns | No concerns | Very low |
| Manual therapy vs Mind-body therapy | 0 | Some concerns | Some concerns | No concerns | No concerns | Major concerns | No concerns | Very low |
| Manual therapy vs Non-invasive brain stimulation | 0 | Some concerns | Some concerns | No concerns | Major concerns | No concerns | No concerns | Very low |
| Manual therapy vs Sham control | 0 | Some concerns | Some concerns | No concerns | No concerns | Major concerns | No concerns | Very low |
| Mind-body therapy vs Non-invasive brain stimulation | 0 | Some concerns | Some concerns | No concerns | Major concerns | No concerns | No concerns | Very low |
| Mind-body therapy vs Sham control | 0 | Some concerns | Some concerns | No concerns | No concerns | No concerns | No concerns | Low |
| Non-invasive brain stimulation vs Usual care | 0 | Some concerns | Some concerns | No concerns | Major concerns | No concerns | No concerns | Very low |

Notes: Direct studies denotes the number of head-to-head trials for the stated comparison. Comparisons with zero direct studies were informed entirely by indirect evidence. CINeMA judgments used a random-effects SMD model and a prespecified equivalence range of −0.20 to 0.20 SMD units.
